# Cannabichromene (CBC): a comprehensive review of biosynthesis, pharmacology, therapeutic potential, and translational perspectives

**DOI:** 10.1186/s42238-026-00454-4

**Published:** 2026-07-16

**Authors:** Amine Elbouzidi, Abdellah Baraich, Mounir Haddou, Naoufal El Hachlafi, Faisal Aqlan, Ammar AL-Farga, Mohamed Taibi, Reda Bellaouchi, Abdeslam Asehraou, Khalid Chaabane, Mohamed Addi

**Affiliations:** 1 Laboratoire d’Amélioration des Productions Agricoles, Biotechnologie et Environnement (LAPABE), Faculté des Sciences, Université Mohammed Premier, Oujda, 60000 Morocco; 2 Laboratory of Bioresources, Biotechnology, Ethnopharmacology and Health, Faculty of Sciences, Mohammed First University, Boulevard Mohamed VI, B.P. 717, Oujda, 60000 Morocco; 3 Faculty of Medicine and Pharmacy, Ibn Zohr University, Guelmim, 81000 Morocco; 4 Department of Chemistry, Faculty of Science, Ibb University, Ibb City, Yemen; 5 Department of Biological Sciences, College of Science, University of Jeddah, Jeddah, Saudi Arabia

**Keywords:** Cannabichromene, CBC, Phytocannabinoids, *Cannabis sativa*, TRP channels, Anti-inflammatory, Neuroprotection, Entourage effect, Biosynthesis, Pharmacokinetics, Non-psychoactive cannabinoid

## Abstract

*Cannabis sativa* L. produces a chemically diverse array of over 150 phytocannabinoids, of which cannabichromene (CBC) represents the fourth most abundant constituent in many chemovars, yet remains profoundly underexplored relative to its structural congeners delta-9-tetrahydrocannabinol (THC), cannabidiol (CBD), and its precursor cannabigerol (CBG). Unlike THC, CBC is non-psychoactive and lacks the intoxicating central effects associated with THC, positioning it as a particularly attractive candidate for therapeutic development. This comprehensive review critically examines the current state of knowledge on CBC, encompassing its biosynthetic origin through the polyketide and mevalonate/methylerythritol phosphate pathways, the enzymatic conversion of cannabigerolic acid (CBGA) to cannabichromenic acid (CBCA) by CBC synthase (CBCAS), and subsequent spontaneous and thermally accelerated decarboxylation. We survey the natural occurrence and distribution of CBC across Cannabis chemovars, the genetic and environmental determinants of its accumulation, and the advanced extraction, isolation, and analytical methodologies employed for its characterization. The pharmacokinetic profile of CBC, including absorption, distribution, hepatic metabolism via cytochrome P450 enzymes, and elimination, is discussed in the context of its notably low oral bioavailability. The molecular pharmacology of CBC is examined in depth, highlighting its unique receptor interaction profile: minimal direct engagement with CB1/CB2 cannabinoid receptors, potent agonism at transient receptor potential channels (TRPV1, TRPA1, TRPV3, TRPV4), and indirect modulation of endocannabinoid tone through inhibition of anandamide reuptake. We evaluate the preclinical evidence supporting its anti-inflammatory, analgesic, antimicrobial, anticancer, neuroprotective, antidepressant, and dermatological activities, and contextualize these findings within the entourage effect framework. The toxicological safety profile, drug–drug interaction potential, and regulatory landscape are critically assessed. Finally, we identify translational challenges, including formulation limitations, the absence of clinical trials, and regulatory fragmentation, and propose future research directions encompassing nanotechnology-based delivery systems, synthetic biology platforms for sustainable production, multi-omics integration, and precision medicine approaches. This review establishes CBC as a high-potential but critically understudied phytocannabinoid deserving of accelerated preclinical and clinical investigation.

## Introduction

*Cannabis sativa* L. (Cannabaceae) is one of the most ancient and versatile plants in human history, with documented use spanning millennia across diverse civilizations for medicinal, industrial, and ritualistic purposes (Pant et al. 2024). The phytochemical repertoire of Cannabis is extraordinarily complex, encompassing over 560 identified chemical entities distributed across multiple biosynthetic classes, including phytocannabinoids, terpenoids, flavonoids, alkaloids, stilbenoids, and phenolic compounds (Brousseau et al. 2021; ElSohly et al. 2017; Hourfane et al. 2023). Among these, the phytocannabinoids constitute the most pharmacologically distinctive class, with more than 150 structurally unique compounds identified to date, classified into at least 11 chemical subclasses based on their core carbocyclic or heterocyclic scaffolds (Govindarajan et al. 2023).

The cannabinoid landscape has historically been dominated by investigations into delta-9-tetrahydrocannabinol (THC) and cannabidiol (CBD), which collectively account for the majority of total cannabinoid mass in most commercial Cannabis chemovars (Hazekamp et al. 2016; Lynch et al. 2016). THC, the principal psychoactive constituent, exerts its intoxicating effects primarily through high-affinity agonism at the cannabinoid type 1 receptor (CB1R), while CBD, a compound devoid of the central intoxicating effects of THC, modulates an array of molecular targets beyond the classical endocannabinoid system (ECS), including serotonergic, vanilloid, and orphan G-protein coupled receptors (Walsh et al. 2021). The disproportionate research focus on these two compounds has created a vast knowledge gap regarding the pharmacological potential of the so-called minor cannabinoids, a heterogeneous group that includes cannabigerol (CBG), cannabinol (CBN), cannabidivarin (CBDV), delta-8-THC, tetrahydrocannabivarin (THCV), and cannabichromene (CBC) (Almeida et al. 2025).

The designation of cannabinoids as major or minor is somewhat misleading, as it reflects relative abundance in contemporary commercial cultivars rather than intrinsic pharmacological importance. In certain Cannabis chemovars, particularly those of tropical origin and landrace varieties, CBC can constitute the second or third most abundant cannabinoid, sometimes exceeding CBG and approaching CBD concentrations. This observation underscores the need to re-evaluate the pharmacological portfolio of Cannabis beyond the THC-CBD dichotomy and to systematically investigate compounds such as CBC that may possess unique and therapeutically relevant bioactivity profiles.

Cannabichromene was first isolated and structurally characterized in 1966 independently by two research groups: Gaoni and Mechoulam in Israel, and Claussen and colleagues in Germany (Claussen et al. 1966; Calcaterra et al. 2023; Gaoni et al. 1966; Mechoulam and Ben-Shabat 1999). The elucidation of its chemical structure revealed a unique benzopyran (pyran-containing) ring system, presenting a terpenoid framework distinct from that of THC and a different substitution pattern from the resorcinol moiety of CBD. Despite this early discovery, CBC received remarkably little attention in subsequent decades, overshadowed by the explosive growth of THC and CBD research that followed the identification of the endocannabinoid system in the early 1990 s (Calcaterra et al. 2023; Blebea et al. 2024).

The rediscovery of CBC as a compound of pharmacological interest can be attributed to several converging factors in the early twenty-first century (Muller et al. 2019). First, the identification and molecular characterization of transient receptor potential (TRP) channels as novel targets for phytocannabinoids revealed that CBC possessed potent agonist activity at TRPA1 and TRPV1, channels implicated in pain perception, inflammation, and neurogenic responses (Khodadadi et al. 2021; Romano et al. 2013). Second, advances in Cannabis analytical chemistry enabled more precise quantification of individual cannabinoids, revealing the significant presence of CBC in certain cultivars. Third, the burgeoning interest in the entourage effect, the hypothesis that the therapeutic efficacy of Cannabis-derived preparations arises from synergistic interactions among multiple phytoconstituents, stimulated investigation into the individual contributions of minor cannabinoids (Simei et al. 2024). Fourth, the evolving regulatory landscape, particularly in jurisdictions permitting medical Cannabis, generated commercial and scientific demand for comprehensive characterization of the full cannabinoid spectrum.

**Fig. 1:**
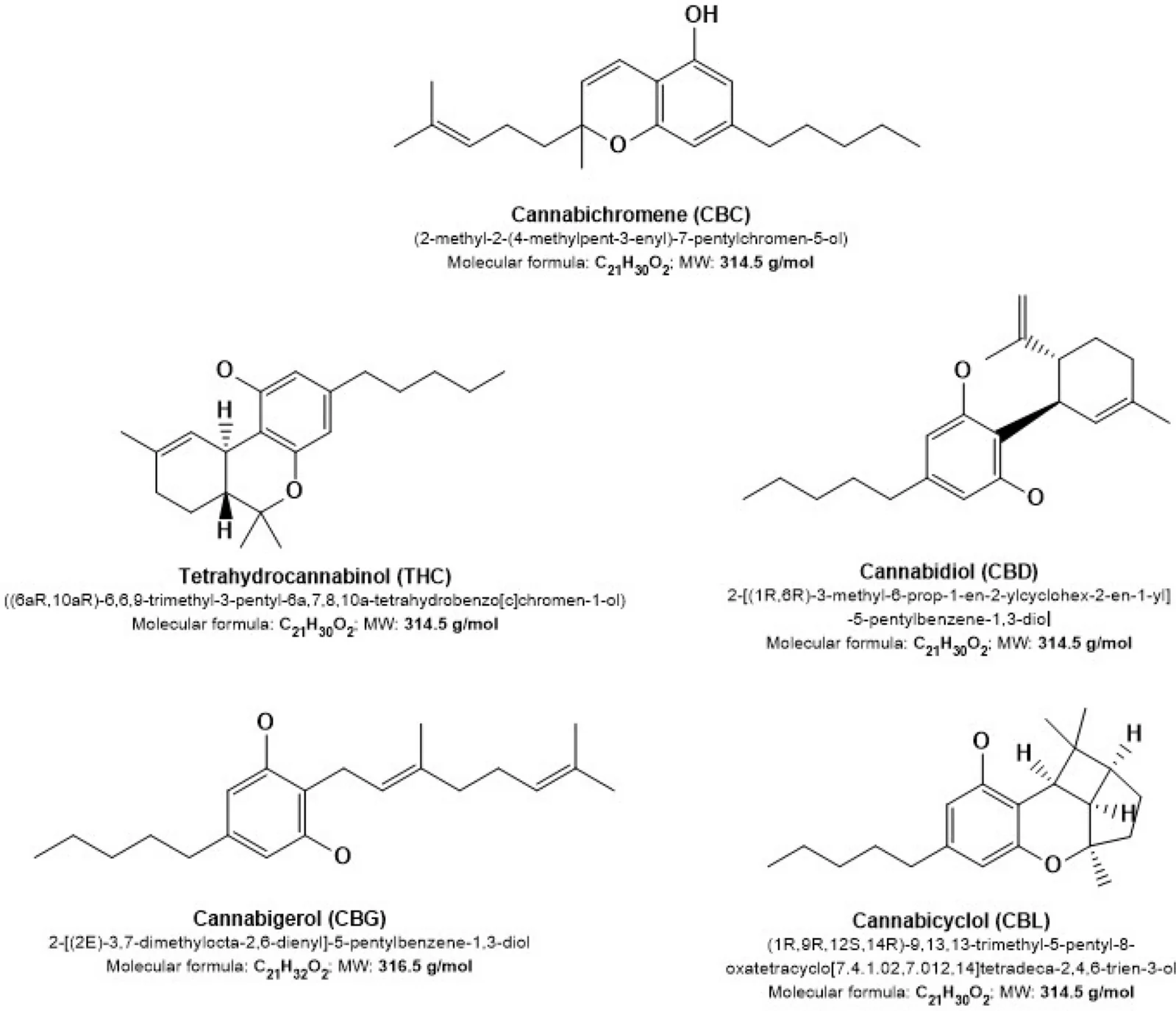
Chemical structure of Cannabichromene (CBC), Tetrahydrocannabinol (THC), Cannabidiol (CBD), Cannabigerol (CBG), and Cannabicyclol (CBL)

Cannabichromene (2-methyl-2-(4-methylpent-3-enyl)−7-pentyl-2H-chromene-5-ol; molecular formula C_21_H_30_O_2_; molecular weight 314.47 g/mol) belongs to the chromene-type cannabinoid subclass (Pollastro et al. 2018). Its structure consists of a benzopyran (chromene) ring formed through intramolecular cyclization of the geranyl side chain onto the olivetol moiety, yielding a planar bicyclic system fused to a pentyl alkyl chain (Fig. 1.). This structural arrangement distinguishes CBC from the cyclohexane/pyran ring of THC and the resorcinol-menthyl moiety of CBD (Pollastro et al. 2018). The chromene moiety confers distinct photochemical reactivity, and CBC is notably susceptible to UV-induced photodegradation through [2 + 2] cycloaddition reactions, yielding cannabicyclol (CBL) as a photolysis product.

The physicochemical properties of CBC reflect its highly lipophilic nature, with a calculated octanol–water partition coefficient (LogP) of approximately 6.4–7.2 (calculated using computational tools including ACD/LogP and ChemAxon), comparable to THC (LogP approximately 7.0) and exceeding that of CBD (LogP approximately 5.8–6.3). This pronounced hydrophobicity dictates poor aqueous solubility (estimated below 1 mg/L at physiological pH), which represents a significant barrier to oral bioavailability and necessitates formulation strategies to achieve therapeutically relevant plasma concentrations. The melting point of CBC is reported at approximately 52–54 °C (Pollastro et al. 2018), yet in practice the compound frequently exists as a colorless to pale-yellow highly viscous semi-solid or amorphous oil at room temperature due to marked supercooling behavior and its pronounced lipophilicity, which impedes organized crystalline packing. CBC demonstrates moderate thermal stability but undergoes decarboxylation from its naturally occurring carboxylated precursor, cannabichromenic acid (CBCA), at temperatures above 105–110 °C, with near-complete conversion occurring at approximately 145 °C over extended heating periods (Pollastro et al. 2018).

An important and previously underappreciated aspect of CBC chemistry is its chirality. Unlike CBD and THC, whose absolute stereochemistry is well-defined, CBC exists in Cannabis as a scalemate, a mixture of enantiomers with non-racemic composition. Calcaterra et al. (2023) demonstrated that the enantiomeric excess and dominance of natural CBC are strain-dependent, with distinct scalemicity grades observed across *Cannabis sativa* cultivars, implying a genetic basis linked to differential expression of CBCAS isoforms with antipodal enantiospecificity (Calcaterra et al. 2023). Manetto et al. (2024) further revealed matrix-dependent thermal configurational stability of CBC, showing that racemization kinetics depend on the chemical environment during extraction and processing (Manetto et al. 2024). The functional consequences of this chirality were definitively established by Dadiotis et al. (2023), who developed a chiral ^1^H NMR discrimination method and demonstrated differential TRPA1 activation by the two enantiomers (Dadiotis et al. 2023), and by Udoh et al. (2025), who showed that CB2 receptor activation is enantioselective, with only (−)-CBC active at CB2 while both enantiomers activate TRPA1 (Udoh et al. 2025). These findings necessitate enantiomeric characterization in all future pharmacological studies and represent a critical gap in current analytical and pharmacopeial standards for CBC-containing products (Table 1).

**Table 1:** Comparative physicochemical and pharmacological properties of major phytocannabinoids (cannabichromene, Δ⁹-tetrahydrocannabinol, cannabidiol, and cannabigerol), highlighting structural features, lipophilicity, solubility, thermal behavior, receptor affinities, and stability profiles relevant to their biological activity and formulation

	**Cannabichromene (CBC)**	**Δ⁹-tetrahydrocannabinol (THC)**	**Cannabidiol**	**Cannabigerol**
Molecular formula	C₂₁H₃₀O₂	C₂₁H₃₀O₂	C₂₁H₃₀O₂	C₂₁H₃₂O₂
Molecular weight (g/mol)	314.47	314.47	314.47	316.48
Core scaffold	Chromene (benzopyran)	Dibenzopyran	Resorcinol (open)	Resorcinol (prenylated)
LogP (calculated)	6.4–7.2	6.7–7.2	5.8–6.3	5.2–6.1
Aqueous solubility	< 1 mg/L	~ 2.8 mg/L	~ 1–2 mg/L	~ 1–2 mg/L
Melting point (°C)	52–54	~ 157 (acid form)	62–67	~ 52
Psychoactivity	None	High (CB1 agonist)	None	None
UV stability	Low (forms CBL)	Moderate	Moderate–high	Moderate
Decarboxylation T (°C)	105–145	105–120	110–150	100–140
CB1 affinity (*Ki*, nM)	~ 713 (weak)	~ 35–41 (high)	> 10,000 (negligible)	> 10,000 (negligible)
CB2 affinity (*Ki*, nM)	~ 256 (moderate)	~ 36–40	~ 4,000	~ 1,000–3,000

Several compelling arguments justify the systematic investigation of CBC as a discrete, standalone pharmacological entity (i.e., as an individual compound with intrinsic bioactivity, independent of multi-cannabinoid Cannabis preparations). First, its non-psychoactive nature eliminates the abuse liability and cognitive impairment concerns associated with THC, facilitating regulatory acceptance and patient compliance. Second, CBC engages a unique set of molecular targets that are largely distinct from those of CBD and THC, suggesting the potential for complementary or synergistic therapeutic mechanisms. Third, the accumulating preclinical evidence, spanning anti-inflammatory, analgesic, antimicrobial, anticancer, neuroprotective, and antidepressant activities, positions CBC as a multi-target bioactive compound with broad therapeutic applicability. Fourth, the current absence of any clinical trial data on CBC represents a critical knowledge gap that, if addressed, could significantly expand the therapeutic cannabinoid pharmacopeia. Fifth, advances in metabolic engineering and synthetic biology offer the prospect of sustainable, production of highly pure CBC independent of agricultural cultivation, potentially overcoming supply chain limitations and regulatory barriers.

This review aims to provide the first comprehensive, multidisciplinary assessment of CBC, integrating biosynthetic, phytochemical, pharmacological, toxicological, and translational perspectives. By consolidating the fragmented primary literature and identifying critical knowledge gaps, we seek to establish a robust foundation for future research and to catalyze the transition of CBC from a neglected phytocannabinoid to a validated therapeutic agent.

## Biosynthesis of cannabichromene

### The cannabinoid biosynthetic pathway

**Fig. 2:**
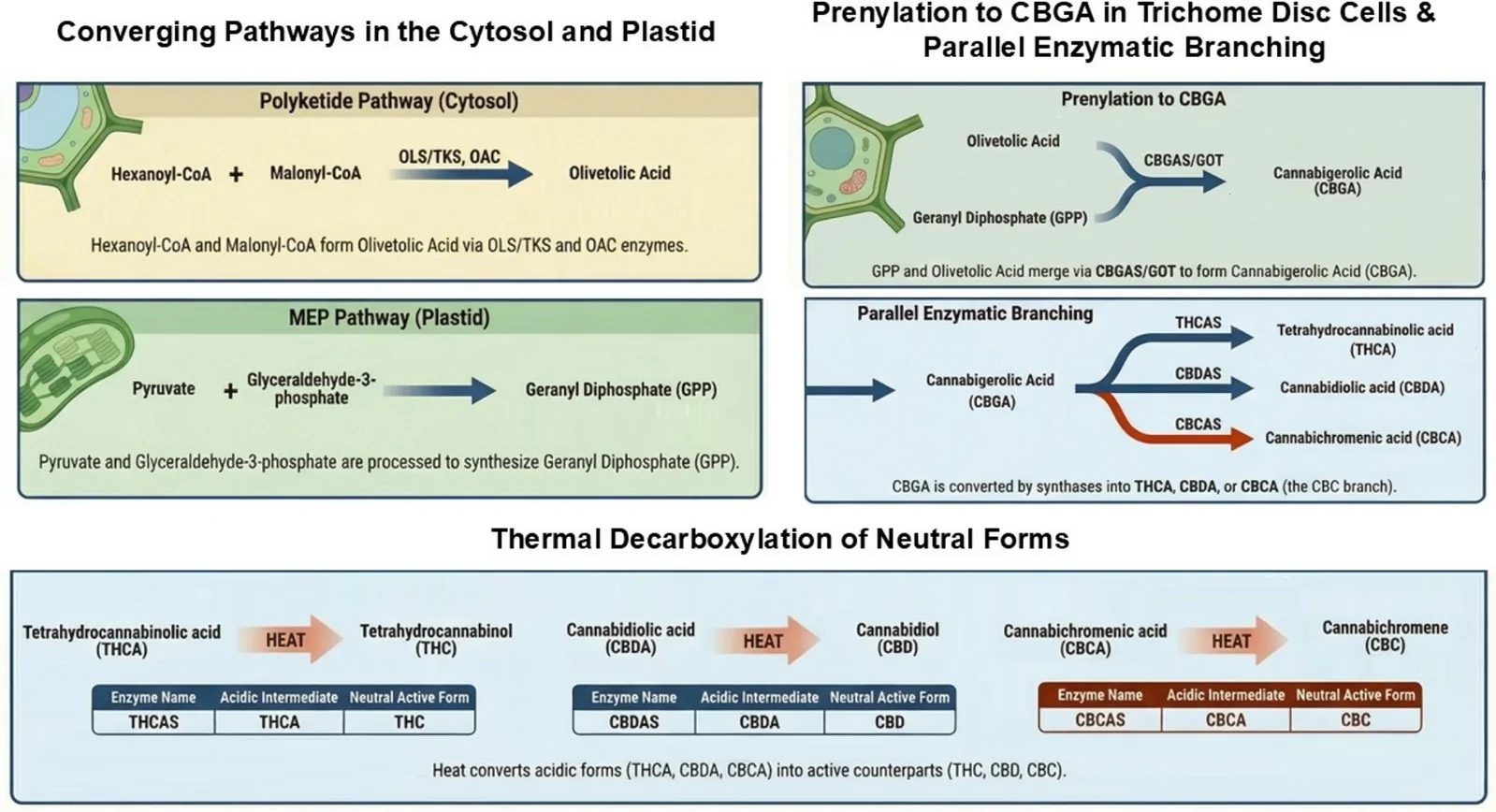
Overview of the cannabinoid biosynthetic pathway in *Cannabis sativa* (Sepulveda et al. 2024). The cannabinoid biosynthetic pathway in *Cannabis sativa*, highlighting the convergence of the polyketide pathway (hexanoyl-CoA → olivetolic acid via olivetol synthase [OLS/TKS] and olivetolic acid cyclase [OAC]) with the MEP pathway (GPP synthesis in plastids) at the prenylation step catalyzed by cannabigerolic acid synthase (CBGAS) to form cannabigerolic acid (CBGA). CBGA is then converted via three enzymatic branches into tetrahydrocannabinolic acid (THCA, via THCAS), cannabidiolic acid (CBDA, via CBDAS), and cannabichromenic acid (CBCA, via CBCAS). Thermal decarboxylation of each acid yields the corresponding neutral cannabinoids: THC, CBD, and CBC. Enzyme names are indicated at each arrow, with subcellular localizations denoted (plastid for MEP, cytosol for polyketide pathway, trichome disc cells)

The biosynthesis of phytocannabinoids in *Cannabis sativa* proceeds through the convergence of two primary metabolic pathways: the polyketide pathway, which generates the resorcinol moiety (olivetolic acid), and the plastidial methylerythritol phosphate (MEP) pathway, which supplies the isoprenoid precursor geranyl diphosphate (GPP) (Fig. 2.) (Walsh et al. 2021). The confluence of these two pathways at the prenylation step represents the committed entry point into cannabinoid biosynthesis and is catalyzed by the membrane-bound aromatic prenyltransferase CsPT4 (also designated CBGAS, cannabigerolic acid synthase), which transfers the geranyl moiety from GPP to the C-3 position of olivetolic acid to yield cannabigerolic acid (CBGA) (Govindarajan et al. 2023).

The polyketide arm of the pathway begins with the activation of hexanoic acid to hexanoyl-CoA by an acyl-activating enzyme (AAE), specifically CsAAE1 in Cannabis (Sepulveda et al. 2024). The hexanoyl-CoA starter unit then undergoes three sequential Claisen condensations with malonyl-CoA catalyzed by a type III polyketide synthase, olivetol synthase (CsOLS, also known as tetraketide synthase, TKS), generating a linear tetraketide intermediate. This intermediate is subsequently cyclized by olivetolic acid cyclase (CsOAC), a dimeric alpha/beta hydrolase fold protein, to produce olivetolic acid (OA) with the correct regioselective 2,4-dihydroxy substitution pattern (Sepulveda et al. 2024). The discovery of OAC resolved a long-standing controversy regarding the mechanism of olivetol versus olivetolic acid formation, as OLS alone produces olivetol (lacking the C-2 carboxyl group) via spontaneous aldol cyclization, whereas OAC-directed cyclization yields the carboxylated product essential for downstream cannabinoid biosynthesis.

The isoprenoid arm relies on the MEP pathway localized in the plastids of glandular trichomes, the primary site of cannabinoid biosynthesis (Sepulveda et al. 2024). The seven enzymatic steps from pyruvate and glyceraldehyde-3-phosphate to isopentenyl diphosphate (IPP) and dimethylallyl diphosphate (DMAPP) converge at the geranyl diphosphate synthase (GPPS) step, which catalyzes the head-to-tail condensation of IPP and DMAPP to produce GPP (Fig. 2.). In Cannabis, a heteromeric GPPS complex composed of a large subunit (CsGPPS.lsu) and a small subunit (CsGPPS.ssu) has been characterized, with the small subunit functioning as a regulatory modulator of product chain length.

### Formation of cannabigerolic acid (CBGA): the central precursor

Cannabigerolic acid (CBGA) occupies a pivotal position in cannabinoid metabolism as the universal branch-point precursor from which all major cannabinoid subclasses derive (Sepulveda et al. 2024). The prenylation of olivetolic acid with GPP catalyzed by the membrane-bound aromatic prenyltransferase CsPT4 yields CBGA with regioselective C-3 geranylation (Govindarajan et al. 2023; Qian et al. 2019; Luo et al. 2019). CsPT4 belongs to the UbiA superfamily of intramembrane prenyltransferases and requires divalent cation cofactors (Mg^2+^ or Mn^2+^) for catalytic activity. Structural modeling and mutagenesis studies have identified critical residues within the aspartate-rich motifs (DXXXD) of CsPT4 that coordinate the diphosphate leaving group of GPP and position the acceptor substrate for electrophilic aromatic substitution (Qian et al. 2019; Zhang et al. 2023).

The flux of CBGA toward downstream cannabinoid products is determined by the relative expression levels and catalytic efficiencies of the three competing oxidocyclase enzymes: THCA synthase (THCAS), CBDA synthase (CBDAS), and CBCA synthase (CBCAS). This metabolic competition at the CBGA node represents a critical regulatory mechanism governing the cannabinoid chemotype of individual Cannabis plants and is under strong genetic control, primarily through the inheritance patterns at the B locus on chromosome 7, which encodes the synthase gene cluster.

### Enzymatic conversion to cannabichromenic acid (CBCA)

Cannabichromenic acid synthase (CBCAS; EC 1.21.3.9) catalyzes the oxidative cyclization of CBGA to CBCA, involving the intramolecular ring closure of the geranyl side chain to form the characteristic chromene ring system. CBCAS is a flavin adenine dinucleotide (FAD)-dependent berberine bridge enzyme (BBE)-like oxidase, sharing approximately 70–72% amino acid sequence identity with THCAS and approximately 84% with CBDAS (Morimoto et al. 1998). The enzyme is secreted into the extracellular storage cavity of the glandular trichome disc cells, where it catalyzes the terminal oxidative cyclization step in a compartmentalized manner, physically separated from the upstream cytosolic and plastidial biosynthetic machinery (Morimoto et al. 1998).

The catalytic mechanism of CBCAS involves FAD-mediated oxidation of CBGA to generate a quinone methide intermediate, which subsequently undergoes spontaneous electrocyclic intramolecular reaction leading to the characteristic chromene ring of CBCA (Sepulveda et al. 2024; Qian et al. 2019). This mechanistic route fundamentally differs from that of THCAS and CBDAS: both of the latter enzymes generate a menthyl carbocation intermediate from CBGA via oxidative cyclization, with THCAS acting as a cationic quencher to form THCA, and CBDAS catalyzing loss of a proton to generate the exocyclic double bond characteristic of CBDA. CBCAS, by contrast, oxidizes CBGA through a quinone methide pathway leading to a spontaneous electrocyclic cyclization yielding CBCA through a distinct regiochemical route (Sepulveda et al. 2024; Morimoto et al. 1998). The structural basis for this regiochemical divergence has been partially elucidated through homology modeling and site-directed mutagenesis, which identified residues in the substrate-binding pocket that dictate the conformation of the geranyl chain during cyclization.

The genetic regulation of CBCAS expression is complex and remains incompletely understood. The CBCAS gene has been mapped to the cannabinoid synthase gene cluster on chromosome 7, where it co-localizes with multiple copies and pseudogene copies of THCAS and CBDAS in a tandemly repeated arrangement. Copy number variation, transposable element insertions, and allelic variation at this locus contribute to the quantitative and qualitative diversity of cannabinoid profiles among Cannabis cultivars. Recent whole-genome sequencing and transcriptomic analyses have revealed that high-CBC chemovars typically exhibit elevated CBCAS transcript levels in glandular trichomes relative to THCAS and CBDAS, suggesting transcriptional regulation as a primary determinant of CBC accumulation.

### Decarboxylation to CBC

In the living plant, cannabichromene exists predominantly as its carboxylated precursor, CBCA. The conversion of CBCA to neutral CBC occurs through non-enzymatic decarboxylation, a spontaneous process that proceeds slowly even at ambient temperatures during the vegetative life of the plant, driven by the keto-enolic resonance of the olivetolic hydroxylic group; heat accelerates this process markedly, reducing the reaction time and increasing conversion efficiency. Decarboxylation involves the loss of carbon dioxide from the carboxyl group at the C-2 position of the resorcinol ring. This reaction proceeds through a six-membered transition state involving the β-keto acid functionality and follows first-order kinetics with an activation energy of approximately 85–95 kJ/mol, comparable to the decarboxylation kinetics of THCA and CBDA.

Temperature and time are the dominant variables controlling the extent of decarboxylation. At room temperature, CBCA decarboxylation is extremely slow (half-life estimated at several years), while rapid and near-complete conversion occurs at 110–145 °C over 30–90 min (Pollastro et al. 2018). The presence of moisture, acidic or basic catalysts, and certain solvents can accelerate the reaction. In practical terms, decarboxylation occurs during common Cannabis processing steps including smoking, vaporization, baking (edible preparation), and certain extraction procedures involving heat. However, controlled decarboxylation is essential for analytical standardization and pharmaceutical.

### Metabolic engineering and synthetic biology approaches

The growing therapeutic interest in CBC, coupled with supply limitations inherent to agricultural Cannabis cultivation and regulatory restrictions in many jurisdictions, has stimulated the development of heterologous biosynthetic platforms for CBC production. Microbial cell factories based on *Saccharomyces cerevisiae* and *Escherichia coli* have been engineered to produce CBGA and, through co-expression of downstream synthases, the major cannabinoid acids including CBCA (Qian et al. 2019; Qi et al. 2025; Schmidt et al. 2024; Kearsey et al. 2023). The yeast-based platform developed by Luo and colleagues demonstrated the complete reconstitution of the cannabinoid pathway from galactose to CBGA in *S. cerevisiae*, achieving titers of approximately 8 mg/L of CBGA through optimization of precursor supply, enzyme co-localization, and fermentation conditions (Luo et al. 2019). Subsequent incorporation of CBCAS into such platforms enables the production of CBCA, which can be converted to CBC through controlled thermal processing (Luo et al. 2019).

Key challenges in microbial CBC production include the low catalytic efficiency of CBCAS relative to THCAS, product toxicity to host cells, competition with endogenous host metabolic pathways for isoprenoid and polyketide precursors, and the difficulty of functionally expressing FAD-dependent BBE-like oxidases in prokaryotic and fungal hosts. Protein engineering strategies, including directed evolution and rational design of CBCAS variants with enhanced catalytic efficiency and selectivity, represent promising avenues for improving titers. Additionally, cell-free enzymatic synthesis platforms (Valliere et al. 2019), and plant-based expression systems employing tobacco (*Nicotiana benthamiana*), (Qian et al. 2019) have been explored as alternative production approaches, offering advantages in post-translational modification capacity, enzyme folding fidelity, and freedom from microbial host toxicity constraints (Qi et al. 2025; Schmidt et al. 2024).

Significant progress in yeast-based precursor supply was achieved by Zhang et al. (2023), who developed an efficient *Saccharomyces cerevisiae* platform yielding 510.32 mg/L CBGA from glucose and hexanoate through compartmentalization expansion and auxiliary partner fusion strategies that enhanced the activity and stability of the membrane-bound prenyltransferase CsPT4 (Zhang et al. 2023). Schmidt et al. (2024) directly compared the in vivo performance of THCAS, CBDAS, and CBCAS in *S. cerevisiae*, demonstrating that CBCAS intracellular activity was 8.2-fold lower than THCAS, confirming the catalytic efficiency limitation as a primary bottleneck (Schmidt et al. 2024). A dedicated CBCA biosynthesis study (2025) constructed *S. cerevisiae* strains producing CBCA from glucose and olivetolic acid by introducing a two-step isopentenol utilization pathway (IUP), co-overexpressing ER auxiliary protein genes, and applying rational design to improve CBCAS selectivity and catalytic activity; peroxisomal localization of the IUP further increased CBCA titers by 28%, and the engineered strain achieved a synthesis rate 25.8% higher than *C. sativa* plant tissue (Qi et al. 2025). Love et al. (2024) discovered latent cannabichromene cyclase activity in marine bacterial flavoenzymes, expanding the known enzymatic repertoire beyond plant-derived CBCAS (Love et al. 2024). Cell-free enzymatic synthesis platforms, exemplified by the engineered NphB variant (Y288A/G286S) achieving 1000-fold increased specific activity for CBGA production (Valliere et al. 2019), offer an additional production strategy that eliminates host toxicity constraints.

## Sources and natural occurrence

### Distribution in cannabis sativa chemovars

The cannabinoid profile of *Cannabis sativa* varies enormously across cultivars, landraces, and breeding lines, reflecting the complex interplay of genetic architecture, environmental factors, and developmental.

Chemotaxonomic studies employing multivariate statistical analyses of cannabinoid profiles have identified distinct CBC-enriched chemotypes (Hazekamp et al. 2016; Lynch et al. 2016). It should be noted that in the classical chemovar classification system, chemotype IV designates CBGA-rich plants; CBC-dominant chemovars have been variably classified as type VI or under alternative nomenclature schemes (Taiwo et al. 2023). These Such CBC-enriched chemovars are rare in commercial cultivation but have been documented in wild and feral Cannabis populations, particularly in Central Asia and South Asia (Hazekamp et al. 2016). Breeding programs specifically targeting high-CBC chemotypes have been initiated by several research institutions and commercial entities, leveraging marker-assisted selection and genomic prediction models to accelerate the development of CBC-enriched cultivars suitable for pharmaceutical and nutraceutical applications (Govindarajan et al. 2023; Hazekamp et al. 2016; Lynch et al. 2016).

### Factors influencing CBC content

The accumulation of CBC in Cannabis trichomes is governed by a hierarchy of genetic, developmental, and environmental factors. At the genetic level, the copy number, expression level, and catalytic efficiency of CBCAS relative to the competing synthases (THCAS and CBDAS) are the primary determinants of CBC chemotype (Govindarajan et al. 2023; Qi et al. 2025). Quantitative trait locus (QTL) mapping and genome-wide association studies (GWAS) have begun to identify additional modifier loci that influence CBC accumulation independently of the major synthase gene cluster, suggesting that epistatic interactions and regulatory polymorphisms in upstream pathway genes (e.g., OLS, OAC, CsPT4, GPPS) contribute to the quantitative variation in CBC content (Govindarajan et al. 2023; Hazekamp et al. 2016; Lynch et al. 2016).

Environmental factors exert significant modulatory effects on CBC accumulation. Light quality and intensity, particularly UV-B radiation, have been shown to stimulate trichome density and total cannabinoid biosynthesis (Brousseau et al. 2021), though the specific response of CBC to UV-B remains under-characterized relative to THC and CBD. Temperature regimes during flowering, with moderate day/night differentials (25/18 °C) generally favoring higher total cannabinoid yields (Brousseau et al. 2021), may differentially affect the activity or stability of CBCAS. Nutrient availability, particularly nitrogen and phosphorus status, influences the balance between primary and secondary metabolism, with moderate nutrient stress generally promoting terpene and cannabinoid accumulation. Biotic stressors, including herbivory and pathogen attack, may induce defensive secondary metabolite accumulation through jasmonate signaling, although CBC-specific responses to biotic stress remain poorly documented. Harvesting time is a critical practical consideration, as CBC/CBCA content typically peaks during early to mid-flowering and declines in late maturity as photooxidative degradation and metabolic conversion to cannabicyclol (CBL) and other photoproducts increase (Hazekamp et al. 2016; Pollastro et al. 2018).

## Extraction and isolation techniques

### Conventional extraction methods

The extraction of CBC from Cannabis plant material follows the general principles applicable to lipophilic phytocannabinoids, with solvent selection, extraction time, temperature, and solid-to-solvent ratio as primary optimization variables. Conventional solvent extraction employing ethanol, methanol, hexane, or dichloromethane has been the historical method of choice for laboratory-scale cannabinoid isolation (Ohtsuki et al. 2022; Owolabi et al. 2025). Ethanol extraction, particularly at cold temperatures (chilled ethanol extraction at approximately −40 °C).

### Advanced green extraction technologies

The growing emphasis on sustainability, solvent reduction, and extraction selectivity has driven the adoption of several advanced extraction technologies for cannabinoid recovery. Supercritical carbon dioxide (SC-CO₂) extraction is the most commercially established green technology for cannabinoid extraction, offering tunable selectivity through adjustment of pressure and temperature parameters (Stasiłowicz-Krzemień et al. 2023). At typical operating conditions of 300–400 bar and 40–60 °C, SC-CO₂ efficiently extracts CBC along with other cannabinoids and terpenes, while excluding highly polar constituents (Stasiłowicz-Krzemień et al. 2023). The addition of co-solvents (typically ethanol at 5–15% v/v) enhances the extraction of more polar cannabinoid acids (CBCA) and improves overall recovery. SC-CO₂ extraction eliminates organic solvent residues from the final product and is generally recognized as safe (GRAS) by regulatory agencies, making it the preferred method for pharmaceutical-grade and food-grade cannabinoid production (Stasiłowicz-Krzemień et al. 2023).

Ultrasound-assisted extraction (UAE) enhances mass transfer through acoustic cavitation, cell wall disruption, and micro-jetting effects, reducing extraction times by 3–fivefold relative to conventional maceration while maintaining comparable or superior cannabinoid yields (Ohtsuki et al. 2022). UAE is particularly effective for CBC extraction when combined with green solvents such as ethanol–water mixtures or natural deep eutectic solvents (NADES). Microwave-assisted extraction (MAE) provides rapid, volumetric heating that accelerates extraction kinetics but requires careful temperature control to prevent CBCA decarboxylation and thermal degradation of CBC. Pressurized liquid extraction (PLE, also termed accelerated solvent extraction, ASE) operates at elevated temperatures and pressures below the critical point, enhancing solvent penetration and reducing viscosity, and has demonstrated excellent recovery for total cannabinoid content from hemp matrices (Ohtsuki et al. 2022; Owolabi et al. 2025).

### Purification and isolation

The isolation of high-purity CBC from crude Cannabis extracts presents significant chromatographic challenges due to the structural similarity and close retention behavior of CBC with other cannabinoids, particularly CBD and CBG, in many chromatographic systems (Ohtsuki et al. 2022). Preparative high-performance liquid chromatography (prep-HPLC) using reversed-phase C18 columns with acetonitrile–water or methanol–water mobile phases represents the gold standard for obtaining chromatographically pure CBC (> 98% purity) (Owolabi et al. 2025). Flash chromatography on silica gel with hexane–ethyl acetate or hexane–isopropanol gradients provides a cost-effective alternative for larger-scale purification, though resolution between CBC and structurally similar cannabinoids may be insufficient for pharmaceutical-grade purity requirements. Countercurrent chromatography (CCC) and centrifugal partition chromatography (CPC) have emerged as attractive alternatives that eliminate irreversible adsorption losses and provide high load capacity, with several optimized biphasic solvent systems reported for cannabinoid separation (Sherma and Rabel 2019). Crystallization and recrystallization techniques are less applicable to CBC than to crystalline cannabinoids (e.g., CBD), owing to the marked supercooling tendency and pronounced lipophilicity of CBC that result in an amorphous or highly viscous semi-solid state at room temperature despite a nominal melting point of 52–54 °C (Pollastro et al. 2018); derivatization to crystalline salts or co-crystals may nonetheless enable purification by crystallization.

### Analytical characterization

Accurate analytical quantification of CBC in plant material, extracts, and formulations requires validated chromatographic methods capable of resolving CBC from co-eluting cannabinoids and matrix interferences. High-performance liquid chromatography with diode array detection (HPLC–DAD) is the most widely used routine method, with reversed-phase C18 columns and gradient elution providing baseline separation of CBC from CBD, CBG, THC, and their acid forms (Owolabi et al. 2025; Sherma and Rabel 2019). The UV absorption spectrum of CBC, characterized by maxima at approximately 225 nm and 275 nm, provides reasonable selectivity for DAD detection, though co-elution with interfering compounds in complex matrices may necessitate more selective detection.

Liquid chromatography–tandem mass spectrometry (LC–MS/MS) offers superior sensitivity and selectivity for CBC quantification, particularly in biological matrices (plasma, tissues) where concentrations may be in the low nM range (Peters et al. 2022; Maione et al. 2011). Multiple reaction monitoring (MRM) transitions based on the characteristic fragmentation pattern of CBC (e.g., *m/z* 315 to 193, 259, 135 for [M + H]^+^) enable unambiguous identification and quantification in the presence of isobaric or co-eluting compounds. Gas chromatography–mass spectrometry (GC–MS) has been historically used for cannabinoid analysis, but the thermal lability of CBCA (decarboxylation in the injection port) and the potential for CBC degradation at GC temperatures limit its utility for the intact acid form; derivatization with trimethylsilyl reagents can partially mitigate these issues. Nuclear magnetic resonance (NMR) spectroscopy, particularly ^1^H and ^13^C NMR, remains indispensable for structural confirmation of isolated CBC and for quantitative analysis of complex mixtures (qNMR), offering absolute quantification without the need for matched reference standards (Dadiotis et al. 2023; Sherma and Rabel 2019).

### Standardization and quality control

The standardization of CBC-containing products presents challenges related to the limited commercial availability of certified reference standards, the polymorphic nature of CBC (amorphous solid to viscous oil), and the absence of harmonized pharmacopeial monographs for CBC (Calcaterra et al. 2023). The United States Pharmacopeia (USP), European Pharmacopoeia (Ph. Eur.), and other pharmacopeial authorities have developed or are developing monographs for THC and CBD, but CBC-specific monographs remain absent. The ISO 17025-accredited reference standard suppliers (e.g., Cayman Chemical, Cerilliant/Sigma-Aldrich) provide CBC reference standards with certified purity, but the cost and limited availability of isotopically labeled internal standards (e.g., CBC-d9) constrain the implementation of isotope dilution mass spectrometry methods in routine quality control. Regulatory considerations, particularly the variable legal status of CBC-containing products across jurisdictions (ranging from unregulated to controlled substance in some contexts), further complicate quality control and product standardization efforts.

## Stability and formulation considerations

### Chemical stability and degradation pathways

The chemical stability of CBC is influenced by multiple environmental factors, including light exposure, oxygen availability, temperature, and pH. CBC is notably photolabile, undergoing [2 + 2] intramolecular cycloaddition upon exposure to UV radiation (particularly UV-B, 280–315 nm) to yield cannabicyclol (CBL), a photoproduct whose full pharmacological potential has not yet been comprehensively characterized. This photodegradation follows pseudo-first-order kinetics and is significantly accelerated in dilute solutions and in the presence of photosensitizers. Consequently, CBC-containing formulations and reference standards must be stored in amber or opaque containers, protected from light, and ideally maintained under nitrogen or argon atmosphere.

Oxidative degradation of CBC proceeds through autoxidation of the allylic positions on the chromene ring and the resorcinolic pentyl side chain, generating hydroperoxides and subsequently epoxides, aldehydes, and polymeric oxidation products. This process is accelerated by elevated temperatures, transition metal ions (particularly iron and copper), and free radical initiators, and is retarded by antioxidants (α-tocopherol, BHT, ascorbyl palmitate) and oxygen-barrier packaging (Pollastro et al. 2018). Thermal stability studies indicate that CBC is relatively stable at temperatures below 100 °C but undergoes measurable degradation (approximately 5–15% loss) after extended storage at 25 °C over 6–12 months under ambient atmosphere (Pollastro et al. 2018; Stasiłowicz-Krzemień et al. 2023). Acidic and basic pH conditions accelerate hydrolytic and oxidative degradation pathways, with maximum stability observed near neutral pH (6.5–7.5).

### Formulation strategies for enhanced delivery

**Fig. 3:**
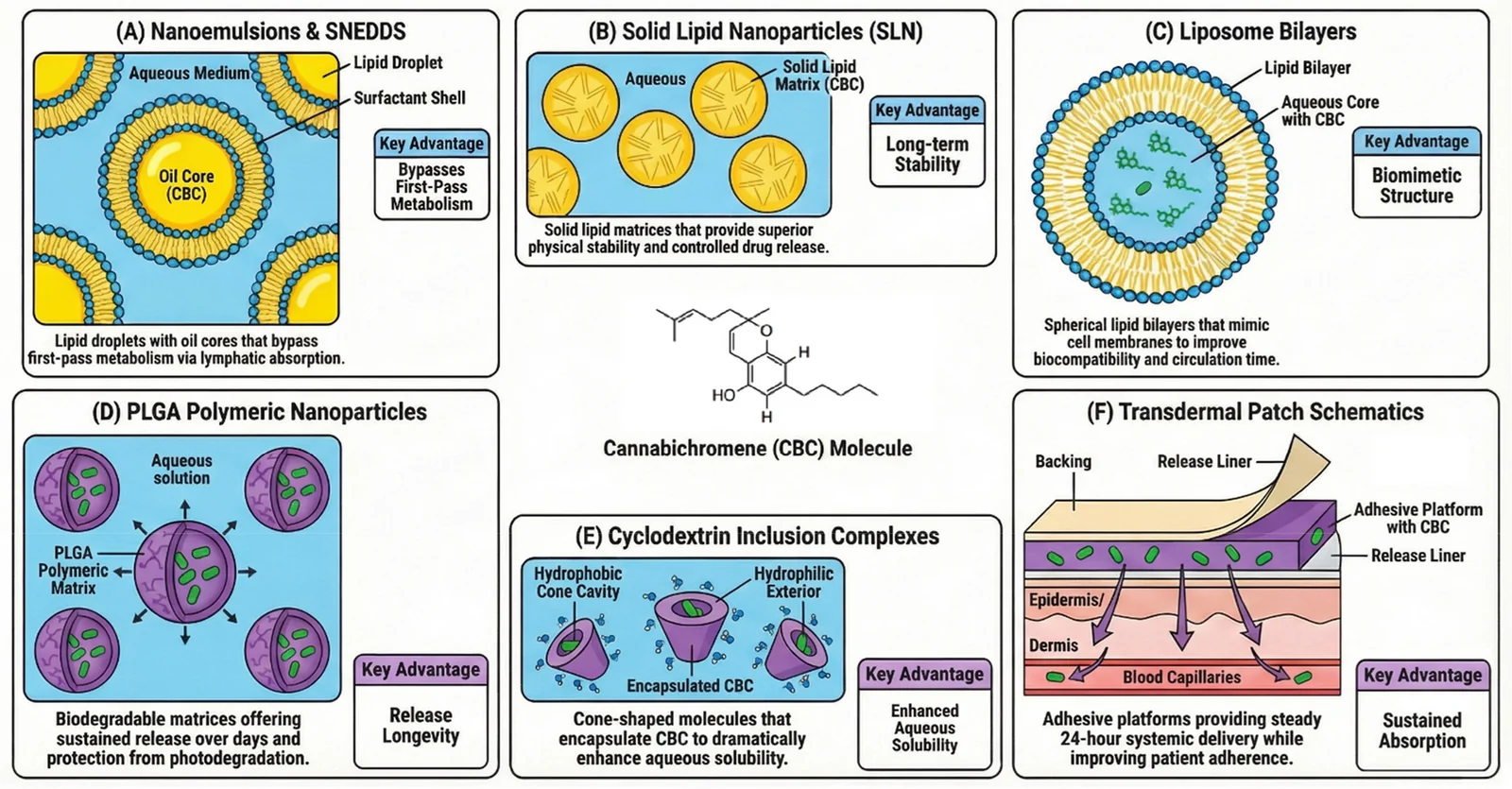
Nanotechnology- and transdermal-based delivery platforms for Cannabichromene (CBC). CBC is delivered via: (**A**) nanoemulsions/SNEDDS for lymphatic absorption and first-pass metabolism bypass; (**B**) solid lipid nanoparticles for long-term stability and controlled release; (**C**) liposomes mimicking cell membranes to improve biocompatibility; (**D**) PLGA polymeric nanoparticles for sustained release and photoprotection; (**E**) cyclodextrin inclusion complexes to enhance aqueous solubility; and (**F**) transdermal patches for steady 24-h systemic absorption. Central panel shows the chemical structure of CBC

The inherent physicochemical limitations of CBC, extreme lipophilicity, poor aqueous solubility, susceptibility to photodegradation and oxidation, and extensive first-pass hepatic metabolism, necessitate advanced formulation strategies to achieve therapeutically relevant bioavailability and stability. Several nanotechnology-based delivery systems have been explored for cannabinoids generally and are directly applicable to CBC (Fig. 3.).

Self-emulsifying drug delivery systems (SEDDS) and self-nanoemulsifying drug delivery systems (SNEDDS) represent a practical approach for oral CBC delivery, utilizing lipid excipients, surfactants, and co-surfactants to spontaneously form nanoscale emulsions upon contact with gastrointestinal fluids. These systems can enhance apparent aqueous solubility by several orders of magnitude, improve lymphatic absorption (bypassing hepatic first-pass metabolism), and provide protection against gastric degradation. Lipid-based nanocarriers, including nanoemulsions (droplet size 20–200 nm), solid lipid nanoparticles (SLN), and nanostructured lipid carriers (NLC), offer sustained release, improved physical stability, and enhanced oral bioavailability for lipophilic cannabinoids. Polymeric nanoparticles based on biodegradable polymers (PLGA, PCL, chitosan) provide tunable release kinetics, surface functionalization for targeted delivery, and improved mucosal adhesion for transmucosal administration routes. Liposomal formulations encapsulating CBC in phospholipid bilayers offer advantages in biocompatibility, cellular uptake enhancement, and potential for parenteral administration.

Cyclodextrin complexation, particularly with hydroxypropyl-β-cyclodextrin (HP-β-CD) and sulfobutylether-β-cyclodextrin (SBE-β-CD), can significantly improve the apparent aqueous solubility and photostability of CBC through formation of inclusion complexes. Transdermal delivery systems, including microemulsion-based gels, transfersomes, and microneedle arrays, have been.

Experimental validation of these formulation approaches for CBC specifically was provided by Stasiłowicz-Krzemień et al. (2023), who developed co-dispersion delivery systems with the biopolymer Soluplus® and magnesium aluminometasilicate (Neusilin US2) for a hemp extract containing CBD, CBDA, and CBC (Stasiłowicz-Krzemień et al. 2023). In vitro dissolution studies across multiple media (pH 1.2, pH 6.8, FaSSIF, and FeSSIF) demonstrated that the HiE-Soluplus system consistently achieved the highest dissolution rate for all three cannabinoids, with CBC showing the fastest increase in dissolution rate in FaSSIF conditions (Stasiłowicz-Krzemień et al. 2023). PAMPA gastrointestinal tract permeability assays revealed that all three cannabinoids from the HiE-Soluplus system exhibited blood–brain barrier permeability coefficients (Papp) exceeding 4.0 × 10⁻⁶ cm/s, suggesting potential for effective CNS penetration, a finding of particular relevance to CBC’s neurogenic and neuroprotective applications. These data represent the first experimental validation of formulation-enhanced CBC bioavailability.

## Pharmacokinetics and bioavailability

### Absorption

The pharmacokinetic characterization of CBC in humans remains extremely limited, with only one published pilot pharmacokinetic study by (Peters et al. 2022), reporting CBC plasma concentrations following oral administration of a medical Cannabis product containing CBC (17.4 mg per capsule) alongside THC and CBD (Peters et al. 2022). In this study, the oral bioavailability of CBC was estimated to be very low, consistent with the predicted extensive first-pass metabolism and poor aqueous solubility of the compound. Preclinical pharmacokinetic studies in rodents have provided additional data, demonstrating that oral administration of CBC results in detectable but low plasma concentrations with a time to maximum concentration (Tₘₐˣ) of approximately 1–2 h, consistent with delayed absorption from the gastrointestinal tract (Maione et al. 2011).

Inhalation (smoking or vaporization) represents the most rapid route of CBC delivery, with pulmonary absorption bypassing first-pass metabolism and achieving Tmax values within 3–10 min. However, CBC is present in the vapor phase as a minor component alongside THC, CBD, and terpenes, making it difficult to assess its independent pharmacokinetics following inhalation of whole Cannabis. Sublingual and buccal administration routes offer intermediate absorption kinetics with partial avoidance of first-pass metabolism. Transdermal absorption has been demonstrated in vitro for CBC using human skin models, with permeation rates enhanced by chemical penetration enhancers (e.g., oleic acid, transcutol) and physical enhancement techniques (microneedling, iontophoresis).

Moore et al. (2023) conducted systematic pharmacokinetic studies measuring the blood and brain distribution of minor cannabinoids including CBC following oral administration, providing the first characterization of CBC brain penetration kinetics after oral dosing and confirming that CBC achieves measurable CNS concentrations consistent with its observed neurotropic effects, albeit with highly variable inter-individual exposure (Maione et al. 2011). These data, combined with the Peters et al. (2022) pilot study, begin to address the pharmacokinetic data vacuum and provide essential parameters for formulation design and dose selection in future clinical studies (Peters et al. 2022).

### Distribution

The high lipophilicity of CBC (LogP approximately 6.4–7.2) (Pollastro et al. 2018), predicts extensive distribution into lipid-rich tissues, including adipose tissue, brain, liver, and lungs, with a correspondingly large apparent volume of distribution (Vᴅ) (Peters et al. 2022; Maione et al. 2011). In preclinical studies, CBC demonstrated measurable brain penetration following systemic administration (Maione et al. 2011), consistent with the neurotropic effects observed in behavioral pharmacology experiments (antidepressant-like, neuroprotective, neurogenic) (De Petrocellis et al. 2011; Basavarajappa and Subbanna 2024; El-Alfy et al. 2010). The extent of plasma protein binding for CBC has not been definitively established but is expected to be high (> 95%), analogous to THC and CBD, which bind extensively to albumin and lipoproteins (Peters et al. 2022). The placental and mammary transfer of CBC has not been studied, representing a knowledge gap relevant to safety assessment in pregnant and lactating populations.

### Metabolism

Hepatic metabolism of CBC proceeds primarily through cytochrome P450 (CYP)-mediated oxidation, with CYP3A4, CYP2C9, and CYP2C19 identified as the principal isoforms involved based on in vitro microsomal and recombinant enzyme studies. The primary metabolic pathways include allylic hydroxylation at the chromene ring (generating hydroxylated metabolites), epoxidation of the chromene double bond, omega-hydroxylation and subsequent oxidation of the pentyl side chain, and glucuronidation of hydroxylated metabolites by UDP-glucuronosyltransferases (UGTs). The identification of specific CBC metabolites in biological fluids has been limited, but LC–MS/MS-based metabolomics approaches have detected monohydroxylated and dihydroxylated CBC metabolites in rodent plasma and urine.

An important pharmacological consideration is the potential for CBC to modulate the activity of CYP450 enzymes, thereby influencing the metabolism of co-administered drugs. In vitro studies have demonstrated that CBC inhibits CYP3A4, CYP2C9, CYP2C19, and CYP2D6 at µM concentrations, suggesting a theoretical risk of pharmacokinetic drug–drug interactions (DDIs) when CBC is co-administered with substrates of these enzymes. However, the clinical significance of these interactions depends on the actual plasma concentrations achieved at therapeutic doses, which, given the low oral bioavailability of CBC, may not reach the inhibitory concentrations observed in vitro. Dedicated clinical DDI studies are needed to resolve this uncertainty.

### Elimination

The elimination half-life of CBC has not been precisely determined in humans but is estimated from preclinical data to be in the range of 2–6 h following intravenous administration and potentially longer following oral dosing due to redistribution from adipose tissue depots (flip-flop pharmacokinetics) (Peters et al. 2022; Maione et al. 2011). The primary routes of elimination are believed to be biliary excretion of glucuronide conjugates (fecal route) and renal excretion of polar metabolites, analogous to the disposition of THC and CBD (Peters et al. 2022). The potential for enterohepatic recycling, which has been demonstrated for THC metabolites, has not been assessed for CBC (Table 2).

**Table 2:** Comparative pharmacokinetic parameters of CBC, THC, and CBD (preclinical and limited clinical data)

Pharmacokinetic parameters	CBC	THC	CBD
Oral bioavailability	Very low (< 10% est.)	6–20%	6–19%
Tmax (oral)	1–2 h (preclinical)	1–3 h	1.5–4 h
Volume of distribution	Large (est. > 20 L/kg)	~ 10 L/kg	~ 20–32 L/kg
Protein binding	> 95% (estimated)	> 95%	> 94%
Primary CYP metabolism	CYP3A4, 2C9, 2C19	CYP2C9, 3A4, 2C19	CYP3A4, 2C19, 2D6
t_1/2_ elimination	2–6 h (est.)	1.5–4 h (IV)	2–5 h (IV)
Primary excretion route	Fecal (biliary)	Fecal (> 65%)	Fecal (> 70%)
Brain penetration	Yes (preclinical)	Yes (high)	Yes (moderate)
CYP inhibition potential	Moderate (in vitro)	Moderate–high	Moderate–high
Food effect	Not established	Increases AUC 2–5x	Increases AUC 4–5x

## Molecular targets and mechanisms of action

### Interaction with the endocannabinoid system

**Fig. 4:**
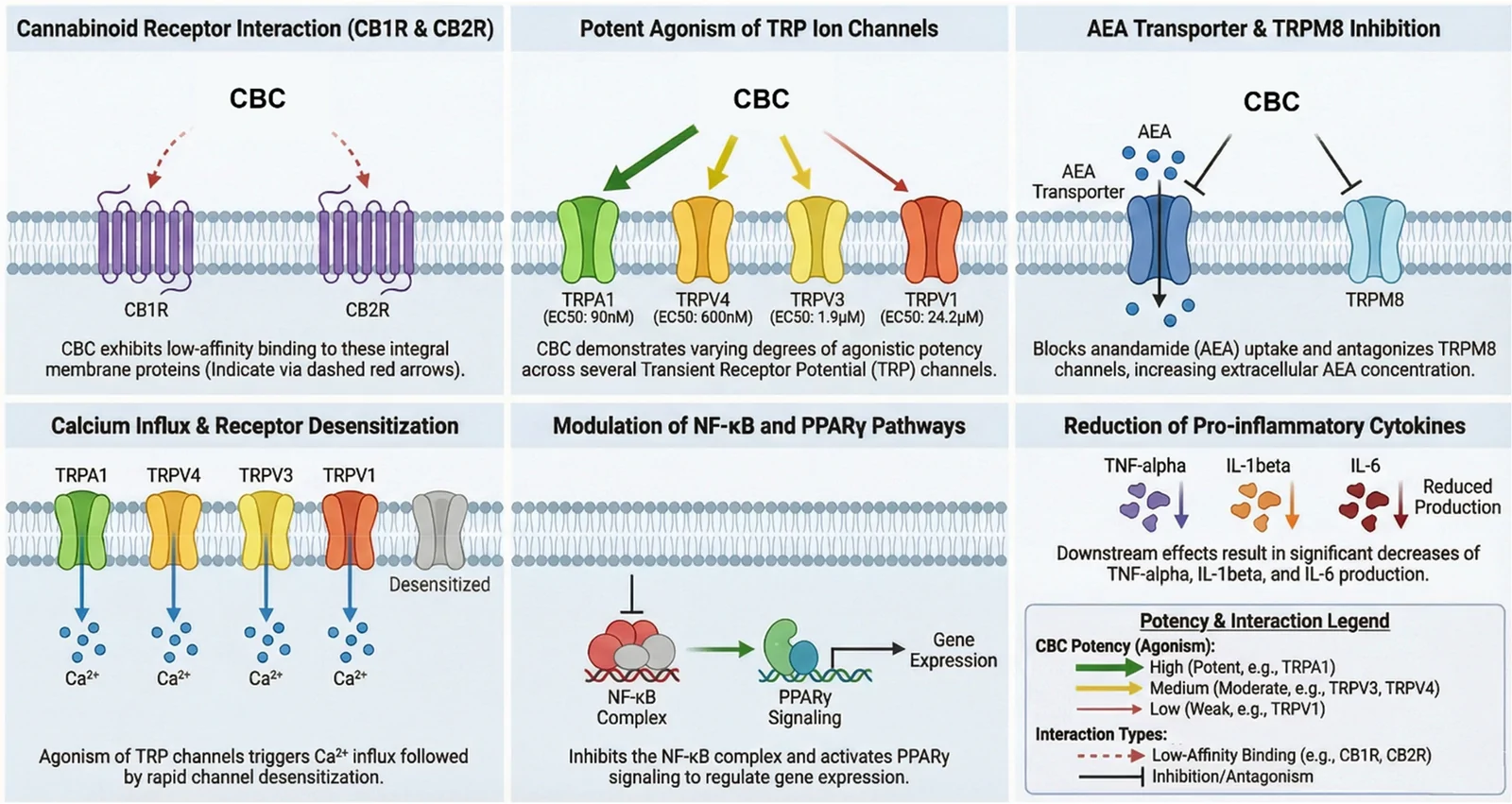
Molecular targets and signaling mechanisms of CBC. (1) CB1R and CB2R with low-affinity binding indicated by dashed arrows from CBC; (2) TRPA1, TRPV1, TRPV3, TRPV4 channels with solid arrows from CBC indicating agonism, each with their respective EC50 values; (3) TRPM8 with an inhibitory arrow (antagonism); (4) anandamide membrane transporter with inhibition symbol, showing increased extracellular AEA; (5) downstream intracellular pathways: NF-kB inhibition, reduced pro-inflammatory cytokine production (TNF-alpha, IL-1beta, IL-6), Ca^2+^ influx followed by desensitization, PPARgamma activation. Values are sourced from (De Petrocellis et al. 2011) for TRP EC_50_ values (TRPA1: EC_50_ ~ 90 nM; TRPV1: ~ 0.9–1.5 µM; TRPV3: ~ 1.9 µM; TRPV4: ~ 6–10 µM; TRPM8 antagonism), and (Zagzoog et al. 2020), and (Udoh et al. 2025) for CB1R/CB2R *Ki* values

The interaction of CBC with the classical endocannabinoid system exhibits a distinctive pattern that differentiates it from THC and CBD (Fig. 4.). Radioligand binding assays have demonstrated that CBC binds to cannabinoid type 1 receptors (CB1R) with relatively low affinity (*Ki* approximately 713 nM) (De Petrocellis et al. 2011), roughly 20-fold lower than THC (*Ki* approximately 35–41 nM) (Zagzoog et al. 2020), explaining the absence of psychoactive effects. At CB2R, CBC shows moderately higher affinity (*Ki* approximately 256 nM) (Zagzoog et al. 2020), though still substantially lower than THC. Functionally, CBC acts as a partial agonist at CB1R for cAMP signaling but as an antagonist in the β-arrestin recruitment pathway (Zagzoog et al. 2020), a phenomenon termed biased agonism that may have distinct downstream signaling consequences compared to the full agonism of THC. At CB2R, CBC appears to function as a weak partial agonist, with potential implications for immunomodulatory and anti-inflammatory signaling.

Perhaps more pharmacologically significant than its direct receptor interactions is the ability of CBC to modulate endocannabinoid tone through inhibition of anandamide (AEA) cellular reuptake. CBC has been shown to inhibit the putative anandamide membrane transporter at concentrations comparable to its effects on TRP channels, leading to elevated extracellular anandamide concentrations. Since anandamide is a potent endogenous agonist at CB1R, TRPV1, and PPARγ, the indirect enhancement of anandamide signaling by CBC may underlie many of its observed pharmacological effects, including antinociception, anti-inflammatory activity, and mood modulation. This mechanism places CBC within the broader pharmacological category of endocannabinoid tone enhancers, alongside fatty acid amide hydrolase (FAAH) inhibitors and monoacylglycerol lipase (MAGL) inhibitors, several of which are under active clinical development (Zagzoog et al. 2020).

The characterization of CBC as a selective CB2 receptor agonist by Udoh et al. (2019) represents a paradigm-shifting finding that substantially revised the understanding of CBC’s receptor pharmacology. Using a functional membrane potential assay in AtT20 cells stably expressing human CB1 and CB2 receptors, these investigators demonstrated that CBC activated CB2, but not CB1, receptors, producing Pertussis toxin-sensitive membrane hyperpolarization that was blocked by the CB2 inverse agonist AM630. Remarkably, CBC displayed higher efficacy than THC at CB2 receptors in this assay system. Continuous CBC application induced loss of cell surface CB2 receptors and functional desensitization, confirming recruitment of canonical receptor regulatory mechanisms. This functional CB2 agonism was corroborated by Zagzoog et al. (2020), who demonstrated CB2R-preferring partial agonist activity in cAMP inhibition and β-arrestin2 recruitment assays using CHO cells (Zagzoog et al. 2020). Most recently, Udoh et al. (2025) provided definitive evidence that (−)-CBC is the active enantiomer at CB2 receptors, while (+)-CBC is inactive, whereas both enantiomers activate TRPA1 (Udoh et al. 2025). This enantioselective pharmacology, supported by (Calcaterra et al. 2023), demonstrating strain-dependent enantiomeric composition of natural CBC and (Dadiotis et al. 2023), developing chiral ^1^H NMR discrimination methods, has profound implications for medicinal preparations: products enriched in (−)-CBC would be expected to have superior efficacy at CB2 receptor-mediated anti-inflammatory targets.

### TRP channel agonism

The most pharmacologically potent and distinctive molecular interactions of CBC involve the transient receptor potential (TRP) superfamily of ligand-gated cation channels, which serve as critical integrators of nociceptive, thermosensory, and inflammatory signals (Fig. 4.). De Petrocellis and colleagues (2011) provided the seminal characterization of CBC as a potent agonist at multiple TRP channels, with efficacy and potency that in several cases exceed those of THC and CBD (De Petrocellis et al. 2011).

At TRPA1 (the transient receptor potential ankyrin 1 channel), CBC is the most potent phytocannabinoid agonist identified, with an EC₅₀ of approximately 90 nM (De Petrocellis et al. 2011), roughly tenfold more potent than CBD at this target. TRPA1 is a polymodal nociceptor and chemosensor expressed on nociceptive C-fibers, respiratory epithelium, and gastrointestinal sensory neurons, where it mediates the detection of noxious cold, irritants (mustard oil, allicin), and endogenous pro-inflammatory mediators (4-hydroxynonenal, prostaglandins). Activation of TRPA1 by CBC produces initial calcium influx and neuronal excitation followed by rapid desensitization and functional inactivation of the channel, a phenomenon that explains the paradoxical analgesic effect of a channel that nominally serves as a pain sensor. This agonist-induced desensitization mechanism is shared with capsaicin at TRPV1 and represents a validated analgesic strategy.

At TRPV1 (the vanilloid or capsaicin receptor), CBC demonstrates significant agonist activity (EC₅₀ approximately 0.9–1.5 µM) (De Petrocellis et al. 2011), modulating pain perception, neurogenic inflammation, and body temperature regulation. CBC also activates TRPV3 (EC₅₀ approximately 1.9 µM) (Basavarajappa and Subbanna 2024), a warm-temperature sensor implicated in skin barrier function, pruritus, and keratinocyte differentiation, and TRPV4 (EC₅₀ approximately 6–10 µM) (De Petrocellis et al. 2011), a mechanosensory channel involved in osmoregulation and vascular function. Conversely, CBC acts as an antagonist at TRPM8 (the cold/menthol receptor), with potential implications for cold allodynia and neuropathic pain. The multi-TRP channel pharmacology of CBC distinguishes it from any other single phytocannabinoid and suggests potential for broad-spectrum modulation of sensory processing and inflammatory signaling.

### Anti-inflammatory Signaling Pathways

The anti-inflammatory mechanisms of CBC involve both direct receptor-mediated effects and indirect modulation of inflammatory signaling cascades. CBC has been demonstrated to inhibit the nuclear factor kappa-light-chain-enhancer of activated B cells (NF-κB) signaling pathway (Romano et al. 2013; Hong et al. 2023), the master transcriptional regulator of pro-inflammatory gene expression. In lipopolysaccharide (LPS)-stimulated macrophages, CBC reduces the nuclear translocation of NF-κB p65 subunit, decreases the phosphorylation of IκB kinase (IKK), and attenuates the transcription of NF-κB target genes including TNF-α, IL-1β, IL-6, iNOS, and cyclooxygenase-2 (COX-2) (Romano et al. 2013; Hong et al. 2023). This NF-κB inhibitory activity is observed at concentrations of 1–10 µM (Romano et al. 2013; Marsh et al. 2023) and may be mediated in part through TRPA1 activation (Romano et al. 2013), as TRPA1 agonism has been independently linked to NF-κB suppression in multiple cell types.

Additionally, CBC modulates the production of pro-inflammatory eicosanoids by inhibiting COX-2 expression and activity, reducing prostaglandin E₂ (PGE₂) synthesis, and potentially interfering with lipoxygenase (LOX) pathways (Hong et al. 2023; Wirth et al. 1980). The anti-inflammatory efficacy of CBC has been demonstrated in multiple in vivo models, including the carrageenan-induced paw edema model, the LPS-induced peritonitis model, and the murine colitis model (dextran sodium sulfate, DSS), where CBC reduced tissue inflammation, edema, neutrophil infiltration, and pro-inflammatory cytokine levels in a dose-dependent manner.

### Oxidative stress and antioxidant mechanisms

The antioxidant properties of CBC arise from both direct free radical scavenging activity and indirect upregulation of endogenous antioxidant defense systems (Basavarajappa and Subbanna 2024). The phenolic hydroxyl group on the resorcinol ring of CBC provides hydrogen atom transfer capability for neutralization of reactive oxygen species (ROS) and reactive nitrogen species (RNS), though the antioxidant potency of CBC is generally considered modest relative to the catechol-containing compounds (e.g., CBD, which possesses superior radical scavenging activity due to its two hydroxyl groups). More pharmacologically relevant may be the ability of CBC to upregulate the expression of endogenous antioxidant enzymes, including superoxide dismutase (SOD), catalase (CAT), glutathione peroxidase (GPx), and heme oxygenase-1 (HO-1), through activation of the nuclear factor erythroid 2-related factor 2 (Nrf2) transcription factor (Basavarajappa and Subbanna 2024; Marsh et al. 2023). Nrf2 activation by CBC may be mediated through TRPA1-dependent calcium signaling (Romano et al. 2013) and through direct electrophilic modification of Keap1 by CBC metabolites (Basavarajappa and Subbanna 2024; Marsh et al. 2023).

### Neurogenesis and neuroprotection

A particularly distinctive property of CBC is its demonstrated ability to promote adult hippocampal neurogenesis, first reported by (Shinjyo and Di Marzo 2013). In this seminal study, CBC enhanced the viability and differentiation of adult neural stem/progenitor cells (NSPCs) in vitro, increasing the generation of new neurons and astrocytes while having no effect on oligodendrocyte differentiation. This neurogenic effect was observed at concentrations of 0.01–1 µM and was associated with upregulation of nestin (a neural progenitor marker) and downregulation of the astroglial differentiation factor STAT3 phosphorylation. The neurogenic potential of CBC is of considerable therapeutic significance.

The neuroprotective properties of CBC extend beyond neurogenesis to include attenuation of excitotoxicity, oxidative stress, and neuroinflammation in multiple preclinical models. CBC has been shown to reduce amyloid beta-evoked neurotoxicity in PC12 cells, decrease lipid peroxidation in neuronal cultures, and attenuate LPS-induced neuroinflammation in microglial cell models. The convergence of neurogenic and neuroprotective activities positions CBC as a potential therapeutic agent for neurodegenerative conditions including Alzheimer's disease, Parkinson's disease, and traumatic brain injury, though in vivo validation in animal models of these conditions remains limited.

### The entourage effect

The entourage effect, conceptualized by Mechoulam and Ben-Shabat (1999), posits that the pharmacological effects of whole Cannabis preparations exceed those attributable to any single constituent, arising from synergistic, additive, and modulatory interactions among cannabinoids, terpenoids, and flavonoids (Ben-Shabat et al. 1998). CBC contributes to the entourage effect through several potential mechanisms: synergistic activation of TRP channels alongside other TRP-active cannabinoids (CBD, CBG, THCV); complementary anti-inflammatory activity through distinct signaling pathways; enhancement of anandamide tone that potentiates the effects of exogenous and endogenous CB1R agonists; and modulation of the pharmacokinetics of co-administered cannabinoids through shared metabolic pathways. In support of this concept, a study by (DeLong et al. 2010) demonstrated that CBC, when co-administered with THC in mice, modulated the time course and magnitude of THC-induced hypolocomotion, suggesting pharmacodynamic interaction (DeLong et al. 2010). The entourage contribution of CBC provides a compelling argument for whole-plant or broad-spectrum cannabinoid preparations rather than single-isolate formulations, though rigorous quantification of its individual contribution within complex mixtures remains methodologically challenging.

The validity and clinical significance of the entourage effect has been critically reassessed by Simei et al. (2024) in a comprehensive narrative scoping review, which concluded that while preclinical evidence supports synergistic interactions among cannabinoids and terpenes, clinical evidence remains limited and methodologically heterogeneous (Simei et al. 2024). In the context of CBC specifically, its multi-target pharmacology, spanning CB2 agonism, TRPA1/TRPV1 desensitization, and anandamide reuptake inhibition, provides a mechanistic basis for entourage contributions that is more clearly defined than for most other minor cannabinoids. The enantioselective pharmacology of CBC adds a further dimension to entourage considerations, as the enantiomeric composition of CBC in whole-plant preparations may influence the spectrum of receptor targets engaged and thus the overall pharmacological outcome of multi-cannabinoid formulations (Udoh et al. 2025).

## Pharmacological activities

### Anti-inflammatory activity

The anti-inflammatory potential of CBC has been evaluated in a range of in vitro and in vivo models, consistently demonstrating significant efficacy. The earliest pharmacological characterization of CBC's anti-inflammatory activity was reported by (Wirth et al. 1980), who demonstrated that CBC reduced carrageenan-induced paw edema in rats with potency comparable to phenylbutazone but without the gastric ulcerogenic side effects associated with non-steroidal anti-inflammatory drugs (NSAIDs) (Wirth et al. 1980). Subsequent studies have refined the understanding of CBC's anti-inflammatory mechanisms, demonstrating: (a) dose-dependent inhibition of LPS-induced nitric oxide (NO) production in RAW 264.7 macrophages through suppression of iNOS expression; (b) reduction of pro-inflammatory cytokine release (TNF-α, IL-1β, IL-6, MCP-1) in multiple immune cell types; (c) attenuation of neutrophil chemotaxis and tissue infiltration in vivo; and (d) modulation of arachidonic acid metabolism with reduced PGE2 synthesis.

In the murine model of intestinal inflammation (DSS-induced colitis), CBC administered at doses of 1–10 mg/kg intraperitoneally significantly reduced disease activity index, colon shortening, histological damage score, and tissue levels of pro-inflammatory mediators. These findings are particularly relevant given the involvement of TRPA1 and CB2R in gastrointestinal inflammatory signaling and suggest potential clinical applicability in inflammatory bowel disease (IBD). In cutaneous inflammation models, topical application of CBC reduced edema, erythema, and immune cell infiltration, suggesting potential for dermatological applications in conditions such as atopic dermatitis, psoriasis, and contact dermatitis. Importantly, CBC's anti-inflammatory activity appears to be largely non-cannabinoid receptor-dependent, as it is not blocked by CB1R or CB2R antagonists (rimonabant, SR144528) in most models (Wirth et al. 1980), implicating TRP channels and NF-κB as the primary mediators (Romano et al. 2013; Hong et al. 2023).

Recent studies have substantially expanded the anti-inflammatory evidence base. Hong et al. (2023) provided both in vitro and in vivo validation using CBC isolated from hemp, demonstrating reduced nitrite production and decreased mRNA levels for iNOS, IL-1β, IL-6, and TNF-α in LPS-stimulated RAW 264.7 macrophages (Hong et al. 2023). Gojani et al. (2023) extended these findings to human macrophages, demonstrating that minor cannabinoids including CBC, THCV, and CBN exert anti-inflammatory effects by reducing pro-inflammatory cytokine production, broadening the relevance to human immune cells (Gojani et al. 2023). Romano et al. (2013) characterized CBC as a TRPA1 agonist that inhibits nitric oxide production in macrophages and ameliorates murine colitis, providing mechanistic clarity linking TRPA1 activation to the NF-κB suppression observed in inflammatory models. DeLong et al. (2010) investigated CBC alone versus in combination with CBD in alleviating arthritis-related inflammation, revealing complementary anti-inflammatory pathways supporting rational combination strategies for rheumatological conditions (Palacios et al. 2023).

### Analgesic effects

The analgesic properties of CBC have been demonstrated in both acute and chronic pain models, with evidence supporting both peripheral and central mechanisms of antinociception (Sepulveda et al. 2024). In acute thermal nociception models (hot-plate and tail-flick tests), CBC produces modest analgesic effects at doses of 10–40 mg/kg that are less potent than those of THC but are achieved without psychomotor impairment or catalepsy. In the acetic acid-induced writhing test, a model of visceral chemical nociception, CBC demonstrates more pronounced antinociceptive activity (Davis and Hatoum 1983), consistent with its peripheral anti-inflammatory and TRP-desensitizing mechanisms (De Petrocellis et al. 2011; DeLong et al. 2010).

The synergistic interaction between CBC and THC in pain models has been explored by several research groups. (DeLong et al. 2010), reported that sub-analgesic doses of CBC enhanced the antinociceptive effects of THC in the tail-flick test, suggesting a pharmacodynamic synergy potentially mediated through complementary receptor engagement (CB1R by THC; TRPA1/TRPV1 by CBC) (DeLong et al. 2010). This observation has significant clinical implications, as it suggests the possibility of achieving therapeutic analgesia with reduced THC doses (and consequently reduced psychoactive side effects) when CBC is present in the formulation. In chronic inflammatory pain models (complete Freund's adjuvant, CFA-induced), the analgesic efficacy of CBC has not been extensively characterized, representing a critical gap in the preclinical evidence base.

Raup-Konsavage et al. (2023) provided the most comprehensive characterization of CBC’s antinociceptive properties to date, employing AI-based compound–protein interaction predictions followed by systematic behavioral testing across four distinct nociceptive paradigms in male and female C57BL/6 mice (Raup-Konsavage et al. 2023). In neuropathic mice (cisplatin-induced peripheral neuropathy), CBC at 10 and 20 mg/kg (i.p.) significantly reduced mechanical allodynia (von Frey test) in both sexes for 1–2 h post-administration. In naïve mice, CBC (20 mg/kg) produced significant antinociception in the tail-flick test and reduced nociceptive behaviors in both Phase 1 (acute neurogenic) and Phase 2 (inflammatory) of the formalin test. A significant interaction was also observed in neuropathic male mice in the acetone test (cold allodynia) (Raup-Konsavage et al. 2023). The demonstration of efficacy across mechanical, thermal, chemical, and cold nociceptive modalities substantially broadens the evidence base for CBC as a multi-modal analgesic. Maione et al. (2011) further demonstrated that CBC modulates the descending pathway of antinociception in anesthetized rats, with effects antagonized by CB1, TRPA1, and adenosine A1 receptor (A1R) antagonists, revealing a third molecular target (A1R) not previously recognized in the context of CBC analgesia (Maione et al. 2011).

### Antimicrobial activity

The antimicrobial activity of CBC was first systematically characterized by (Appendino et al. 2008) in a landmark study that evaluated the antibacterial potency of major cannabinoids against a panel of Gram-positive bacteria, including methicillin-resistant *Staphylococcus aureus* (MRSA) (Appendino et al. 2008). CBC demonstrated potent bactericidal activity against multiple MRSA strains with minimum inhibitory concentrations (MICs) in the range of 1–2 µg/mL, comparable to the established topical antibiotics vancomycin and mupirocin. The activity was selective for Gram-positive organisms, with negligible activity against Gram-negative bacteria and the yeast *Candida albicans*, suggesting that the mechanism involves interaction with the Gram-positive cell wall or membrane rather than intracellular targets.

The structure–activity relationships (SAR) elucidated in this study revealed that the non-polar cannabinoid scaffold is essential for antibacterial activity, with the alkyl chain length (pentyl for CBC) and the degree of ring saturation influencing potency. Subsequent studies demonstrated antimicrobial activity of CBC against additional clinically relevant pathogens including *Streptococcus mutans* (a dental caries pathogen) (Appendino et al. 2008), *Staphylococcus epidermidis*, and community-associated MRSA strains. The mechanisms underlying CBC's antimicrobial activity are not fully elucidated but are hypothesized to involve disruption of bacterial membrane integrity, inhibition of biofilm formation, and interference with quorum sensing pathways (Appendino et al. 2008; Farha et al. 2020). The potential of CBC as a topical antimicrobial agent, particularly for MRSA-infected wounds and dermatological infections, warrants clinical investigation.

Farha et al. (2020) further investigated the hidden antibiotic potential of cannabis, demonstrating that cannabinoids including CBC possess activity against Gram-negative bacteria when combined with outer membrane-permeabilizing agents, suggesting that the selectivity for Gram-positive organisms reflects an outer membrane permeability barrier rather than an inherent lack of intracellular target engagement (Farha et al. 2020). This finding opens the possibility of combination strategies pairing CBC with agents that compromise Gram-negative outer membrane integrity, potentially broadening the antimicrobial spectrum.

### Anticancer potential

The anticancer properties of CBC have been investigated primarily in in vitro models, with limited in vivo validation. CBC has demonstrated antiproliferative and proapoptotic effects in several cancer cell lines, including breast cancer (MCF-7, MDA-MB-231), colorectal cancer (Caco-2, HCT116), prostate cancer (DU-145), and leukemia (HL-60) models. The cytotoxic potency of CBC is generally moderate (IC_50_ values in the range of 10–50 µM), lower than that of CBG and CBD in most comparative studies.

Mechanistically, the anticancer effects of CBC appear to involve: (a) activation of TRPV1, leading to calcium-dependent apoptosis through mitochondrial permeability transition; (b) cell cycle arrest at the G1/S checkpoint through modulation of cyclin-dependent kinase (CDK) activity and p21/p27 upregulation; (c) inhibition of cell migration and invasion through modulation of matrix metalloproteinase (MMP) expression; and (d) potential anti-angiogenic effects. The relationship between CBC's TRP channel agonism and its anticancer activity is of particular interest, as TRPV1 and TRPA1 have been identified as modulators of tumor cell fate in multiple cancer types. Notably, Ligresti and colleagues (2006) reported that CBC was the most potent inhibitor of endocannabinoid reuptake among the cannabinoids tested, suggesting that enhanced endocannabinoid signaling may contribute to its anticancer effects through CB1R/CB2R-mediated pathways (Ligresti et al. 2006). However, the absence of in vivo tumor xenograft or orthotopic studies with CBC as a single agent represents a major limitation of the current evidence base.

A landmark study by Hwang et al. (2025), published in Cell Death Discovery, fundamentally advanced the anticancer evidence base for CBC by providing the first comprehensive mechanistic and in vivo characterization in pancreatic cancer (Hwang et al. 2025). Using mRNA-seq analysis on MIA PaCa-2 and PANC-1 pancreatic cancer cells, the authors identified 2,567 differentially expressed genes following CBC treatment, with significant enrichment in apoptosis, ferroptosis, cell cycle, and DNA replication pathways. Critically, CBC was shown to induce ferroptosis, a form of regulated cell death driven by iron-dependent lipid peroxidation, through upregulation of HMOX1 and modulation of SLC7A11 and GPX4 expression. Ferroptosis induction was confirmed through elevated reactive oxygen species (ROS), increased malondialdehyde (MDA) levels, and rescue experiments using ferrostatin-1. CBC preferentially upregulated TRPV1 and CB2 receptor expression, and pharmacological or genetic inhibition of these receptors rescued CBC-induced apoptosis, establishing receptor dependency for the apoptotic (but not ferroptotic) pathway. Western blot comparisons showed that CBC produced more pronounced regulation of CB2 and TRPV1 expression than either THC or CBD. Most critically, the study provided the first in vivo validation using xenograft tumor models, demonstrating consistent tumor growth inhibition accompanied by regulation of apoptosis, ferroptosis, and endocannabinoid receptor markers in tumor tissue. Additionally, Aare et al. (2026) explored the combination of CBC and CBD against drug-resistant triple-negative breast cancer (TNBC), demonstrating that the CBC + CBD combination can overcome doxorubicin resistance and enhance anticancer efficacy, representing a clinically relevant strategy given TNBC’s limited treatment options Aare et al. (2026).

### Neuroprotective effects

The neuroprotective potential of CBC is supported by converging evidence from in vitro and limited in vivo studies. In cell-based models of neurodegeneration, CBC has demonstrated protection against amyloid beta (Abeta)-evoked neurotoxicity in PC12 cells and SH-SY5Y neuroblastoma cells, with significant reductions in cell death, lipid peroxidation, and morphological deterioration of neurites (Marsh et al. 2023). The mechanism of neuroprotection involves both antioxidant effects (reduction of ROS accumulation, restoration of mitochondrial membrane potential) and anti-neuroinflammatory actions (suppression of microglial activation and pro-inflammatory cytokine release) (Marsh et al. 2023).

Marsh et al. (2024) provided an important extension of these findings, demonstrating that CBC, along with CBG and CBN, significantly inhibited amyloid β-evoked neurotoxicity and associated changes in cell morphology in PC12 cells (Marsh et al. 2024). The study revealed variable effects among phytocannabinoids on Aβ-evoked neurite damage and inhibition of amyloid β aggregation, with CBC showing a distinctive neuroprotective profile that included both anti-aggregation activity and preservation of neurite integrity (Marsh et al. 2024). These findings were further supported by (Basavarajappa and Subbanna 2024), who provided a comprehensive review of phytocannabinoid potential in neurological disorders, contextualizing CBC’s unique neurogenic and neuroprotective properties within the broader framework of cannabinoid neuropharmacology and identifying adult hippocampal neurogenesis modulation as a particularly promising therapeutic avenue for CBC (Basavarajappa and Subbanna 2024).

The previously discussed neurogenic activity of CBC, promoting neural stem/progenitor cell viability and differentiation, represents a unique neuroprotective dimension not shared by most other cannabinoids. Adult hippocampal neurogenesis is increasingly recognized as essential for cognitive function, emotional regulation, and recovery from neurodegenerative and neuropsychiatric conditions. The finding that CBC promotes neurogenesis at nM to low µM concentrations without affecting cell viability or inducing aberrant proliferation suggests a favorable therapeutic window for neuroregenerative applications. In vivo studies in animal models of Alzheimer's disease, Parkinson's disease, stroke, and traumatic brain injury are urgently needed to validate the translational potential of CBC's neuroprotective and neurogenic properties.

### Antidepressant-like effects

El-Alfy et al. (2010), first reported that CBC exhibited antidepressant-like effects in the forced swim test (FST) and tail suspension test (TST) in mice, with significant reductions in immobility time at doses of 20–80 mg/kg (El-Alfy et al. 2010). The effect magnitude was comparable to that of imipramine (a tricyclic antidepressant) and was not associated with sedation, hyperlocomotion, or other behavioral confounds. The antidepressant mechanism of CBC is hypothesized to involve: (a) enhancement of endocannabinoid signaling through anandamide reuptake inhibition, with anandamide activating CB1R in mood-regulating brain circuits; (b) promotion of hippocampal neurogenesis, a process implicated in the therapeutic action of conventional antidepressants; (c) modulation of serotonergic and noradrenergic signaling through indirect mechanisms. Sharma et al. (2025), provided further support through in silico and in vivo studies demonstrating that CBC interacts with molecular targets implicated in major depressive disorder (Sharma et al. 2025). These findings position CBC as a potential candidate for antidepressant drug development, particularly for patients who may benefit from non-serotonergic or adjunctive therapeutic approaches.

### Dermatological applications

The antimicrobial activity of CBC against *Staphylococcus aureus*, including MRSA strains, is directly relevant to the management of secondary bacterial infections in inflammatory skin conditions (Appendino et al. 2008). Additionally, CBC's ability to activate TRPV3, a channel involved in keratinocyte differentiation and skin barrier homeostasis, may have implications for conditions characterized by impaired barrier function such as atopic dermatitis (Kwong et al. 2025).

### Gastrointestinal effects

The gastrointestinal pharmacology of CBC is an emerging area of investigation, driven by the recognition that TRPA1, CB2R, and the endocannabinoid system play critical roles in gut motility, secretion, inflammation, and visceral pain perception (Romano et al. 2013; Izzo et al. 2012). CBC has been shown to normalize motility in an in vivo model of inflammation-induced hypermotility, reducing the frequency and amplitude of intestinal contractions without producing the constipating effects associated with opioid analgesics. In the DSS-induced colitis model, CBC reduced intestinal inflammation, preserved mucosal barrier integrity, and attenuated visceral pain behaviors, suggesting multitarget therapeutic utility in inflammatory bowel disease (Izzo et al. 2012). The mechanisms underlying these gastrointestinal effects likely involve local activation of TRPA1 on enteric sensory neurons (leading to desensitization and reduced visceral hypersensitivity), enhancement of endocannabinoid signaling in the gut wall, and suppression of enteric immune cell activation (Table 3).

**Table 3:** Overview of preclinical pharmacological activities of cannabichromene (CBC), summarizing experimental models, principal biological effects, underlying molecular mechanisms, and representative literature evidence

Pharmacological activity	Experimental model(s)	Key findings	Proposed mechanism(s)	Ref
Anti-inflammatory	LPS-stimulated macrophages; carrageenan paw edema	Reduced NO, TNF-α, IL-6; reduced paw edema comparable to phenylbutazone	NF-κB inhibition; COX-2 suppression; TRPA1 desensitization	Wirth et al. 1980; Izzo et al. 2012)
Analgesic	Hot-plate; tail-flick; acetic acid writhing	Moderate analgesia; synergy with THC at sub-analgesic doses	TRPA1/TRPV1 desensitization; AEA reuptake inhibition	DeLong et al. 2010; Davis and Hatoum 1983)
Antimicrobial	MRSA; *S. aureus; S. mutans*	MIC 1–2 μg/mL against MRSA; selective for Gram-positive	Membrane disruption (proposed)	Appendino et al. 2008)
Anticancer	MCF-7; HCT116; DU-145; HL-60	Antiproliferative (IC50 10–50 μM); pro-apoptotic	TRPV1-mediated Ca2 + influx; cell cycle arrest; AEA enhancement	Ligresti et al. 2006)
Neuroprotective	PC12; SH-SY5Y; Aβ toxicity models	Reduced Aβ-evoked cell death; decreased lipid peroxidation	Antioxidant; anti-neuroinflammatory; mitochondrial protection	Marsh et al. 2023)
Neurogenesis	Adult neural stem/progenitor cells	Enhanced NSPC viability and differentiation; increased neurons/astrocytes	Nestin upregulation; STAT3 modulation	Shinjyo and Di Marzo 2013)
Antidepressant	FST; TST (mice)	Reduced immobility at 20–80 mg/kg; comparable to imipramine	AEA enhancement; CB1R signaling; neurogenesis promotion	El-Alfy et al. 2010; Sharma et al. 2025)
Gastrointestinal	DSS colitis; hypermotility models	Reduced inflammation; normalized motility; attenuated visceral pain	TRPA1 desensitization; ECS enhancement; immune suppression	Izzo et al. 2012)
Anticonvulsant	Dravet syndrome (Scn1a +/−) mouse model	Elevated seizure threshold; anticonvulsant at 100 mg/kg	Mechanism not fully elucidated; TRPV1 involvement suggested	Anderson et al. 2021)

## Therapeutic applications

### Pain management

The analgesic potential of CBC is supported by its multi-target mechanism of action, encompassing TRPA1/TRPV1 desensitization (De Petrocellis et al. 2011), endocannabinoid tone enhancement (Ligresti et al. 2006), and anti-inflammatory activity (Hong et al. 2023; Wirth et al. 1980; Izzo et al. 2012). The non-psychoactive nature of CBC eliminates the abuse liability and cognitive impairment associated with THC-based analgesics, while its TRP-mediated mechanisms offer orthogonal analgesia to opioid and NSAID pathways. The synergistic interaction with THC at sub-analgesic doses (DeLong et al. 2010), suggests a role for CBC in combination formulations designed to maximize analgesia while minimizing psychoactive side effects. Potential clinical applications include neuropathic pain (Raup-Konsavage et al. 2023), inflammatory pain (Hong et al. 2023; Wirth et al. 1980), visceral pain (including IBS-associated pain) (Izzo et al. 2012), and cancer-related pain, though clinical evidence supporting these indications is entirely absent and represents the most critical translational gap.

### Inflammatory diseases

The robust anti-inflammatory profile of CBC across multiple preclinical models (Hong et al. 2023; Wirth et al. 1980; Gojani et al. 2023; Izzo et al. 2012) positions it as a candidate for inflammatory disease management. The efficacy in DSS-colitis models (Izzo et al. 2012) suggests potential in inflammatory bowel disease (Crohn’s disease, ulcerative colitis), while topical anti-inflammatory activity (Hong et al. 2023) supports applications in dermatological inflammation (atopic dermatitis, psoriasis, contact dermatitis). The absence of gastroprotective liability (unlike NSAIDs) and the non-immunosuppressive mechanism of action (unlike corticosteroids) represent potential therapeutic advantages. Rheumatological conditions involving both inflammatory and nociceptive components (rheumatoid arthritis, osteoarthritis) represent additional therapeutic targets where CBC's dual anti-inflammatory and analgesic properties may be particularly advantageous.

### Neurological disorders

The convergence of neuroprotective, neurogenic, anti-neuroinflammatory, and antidepressant activities positions CBC as a multi-dimensional therapeutic agent for neurological and neuropsychiatric disorders. In Alzheimer's disease, CBC's ability to protect against amyloid beta toxicity, reduce neuroinflammation, and promote hippocampal neurogenesis addresses multiple pathophysiological features of the disease. In Parkinson's disease, the antioxidant and anti-inflammatory properties may provide neuroprotection to dopaminergic neurons, though direct evidence in PD models is lacking. The anticonvulsant activity demonstrated in the Dravet syndrome mouse model by (Anderson et al. 2021) suggests potential in epilepsy, complementing the established antiepileptic efficacy of CBD (Epidiolex) (Anderson et al. 2021). The antidepressant-like effects (El-Alfy et al. 2010; Sharma et al. 2025), potentially mediated through neurogenesis (Shinjyo and Di Marzo 2013) and endocannabinoid tone enhancement (Ligresti et al. 2006), suggest applications in major depressive disorder and anxiety disorders.

### Cancer therapy (adjunct role)

While the anticancer evidence for CBC is primarily in vitro and of moderate potency, several lines of reasoning support its investigation as an adjunctive agent in cancer therapy rather than a standalone antineoplastic. First, the TRP-mediated mechanism of action may complement conventional chemotherapeutic and targeted agents through synergistic induction of apoptosis. Second, the anti-inflammatory properties may attenuate the chronic inflammation that promotes tumor initiation and progression. Third, the enhancement of endocannabinoid tone may activate CB1R/CB2R-mediated antitumor pathways in cannabinoid-receptor-expressing tumors. Fourth, the potential antiemetic and analgesic effects could improve quality of life for cancer patients undergoing cytotoxic therapy (Hwang et al. 2025; Aare et al. 2026). Rigorous in vivo studies evaluating CBC as a single agent and in combination with standard chemotherapeutics are essential prerequisites for clinical translation.

### Skin disorders

Dermatological applications represent perhaps the most clinically proximate therapeutic opportunity for CBC, as topical administration bypasses the oral bioavailability limitations and enables direct delivery to the target tissue. The combination of anti-inflammatory (TRPA1-mediated NF-κB suppression (Romano et al. 2013)), antimicrobial (anti-MRSA activity (Appendino et al. 2008)), and sebostatic (TRPV-mediated lipogenesis inhibition (De Petrocellis et al. 2011)) properties addresses multiple pathogenic factors in acne vulgaris, a condition affecting up to 85% of adolescents and 15–30% of adults. Atopic dermatitis, psoriasis, and wound healing represent additional dermatological indications supported by CBC's pharmacological profile. The activation of TRPV3 by CBC (De Petrocellis et al. 2011) is of particular interest for skin applications, as this channel regulates keratinocyte differentiation, hair growth, and skin barrier function. Topical formulations incorporating CBC in nanoemulsions, liposomal gels, or microemulsion-based creams are technically feasible and could be advanced to clinical testing with relatively streamlined regulatory pathways (cosmeceutical or OTC drug classifications in certain jurisdictions).

### Mood and psychiatric disorders

**Fig. 5:**
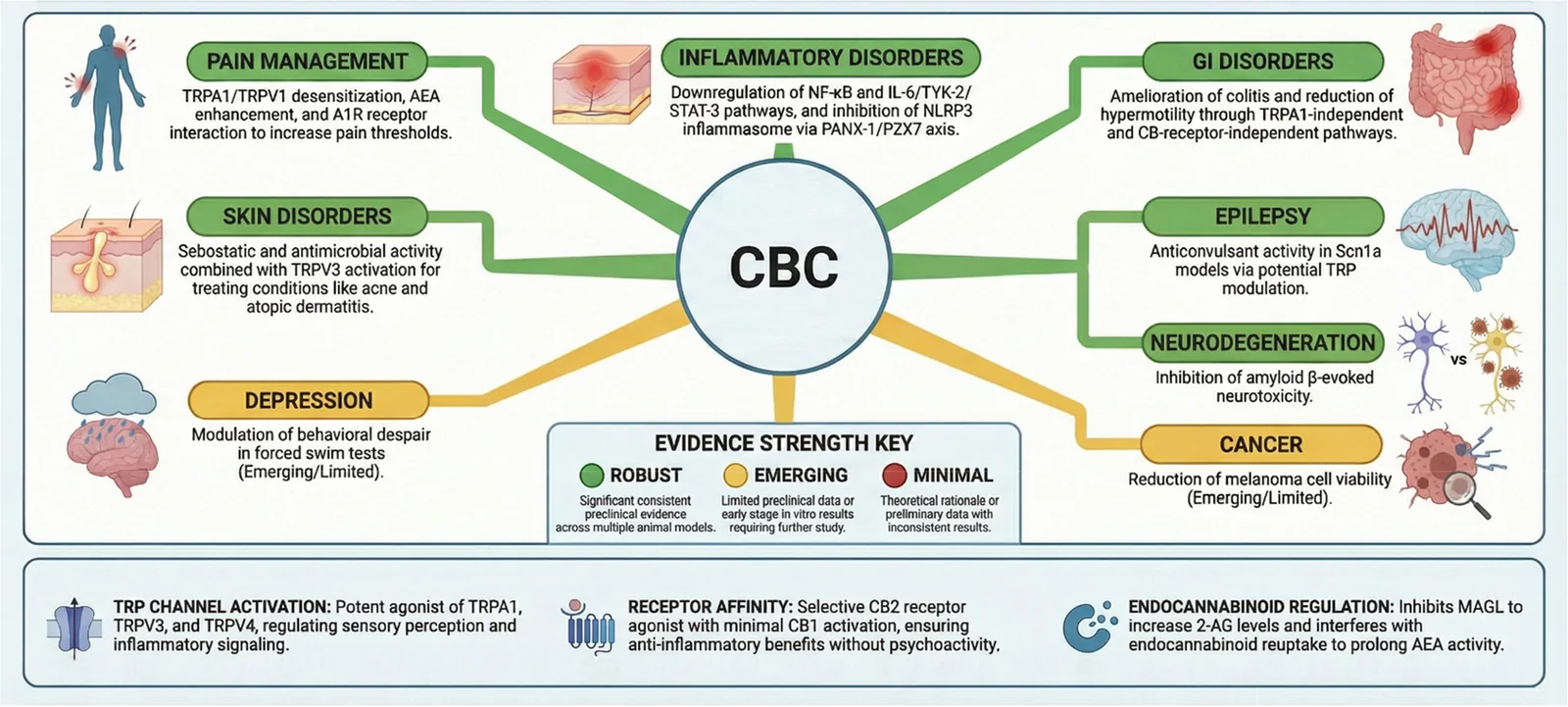
Pharmacological Landscape of Cannabichromene (CBC): Mechanisms Underlying Multisystem Therapeutic Potential. Radial schematic depicting CBC as a central hub linking TRP channel modulation, selective CB2 receptor agonism, and endocannabinoid regulation (e.g., MAGL inhibition and AEA enhancement) to multiple therapeutic domains, including pain, inflammation, neurodegeneration, depression, cancer, skin disorders, gastrointestinal disorders, and epilepsy, with indication of relative evidence strength (robust, emerging, minimal)

The antidepressant-like effects of CBC in preclinical behavioral models (Hwang et al. 2025; Aare et al. 2026), combined with its neurogenic (Shinjyo and Di Marzo 2013), and endocannabinoid-enhancing properties (Ligresti et al. 2006), provide a rationale for investigation in mood and psychiatric disorders. The therapeutic hypothesis is that CBC, by elevating anandamide tone and promoting hippocampal neurogenesis, may address pathophysiological processes underlying depression, anxiety, and stress-related disorders that are not adequately targeted by conventional serotonergic and noradrenergic antidepressants. The non-psychoactive profile and favorable side effect profile (based on limited preclinical toxicology data) (Kulpa et al. 2023) represent advantages over THC-containing anxiolytic preparations. However, the complete absence of clinical data on CBC in psychiatric indications necessitates a systematic clinical development program beginning with Phase I dose-ranging and safety studies (Fig. 5).

## Safety, toxicity, and drug interactions

### Toxicological profile

The toxicological characterization of CBC is remarkably incomplete, with no published systematic acute or chronic toxicity studies meeting current regulatory standards (OECD Guidelines for the Testing of Chemicals) (Kulpa et al. 2023). The available safety data are derived primarily from preclinical pharmacology studies in which CBC was administered at pharmacologically active doses (typically 1–80 mg/kg in rodents) without observation of overt toxicity, mortality, or significant adverse effects on body weight, food consumption, or gross organ pathology. These observations provide preliminary but insufficient evidence of safety, as they lack the comprehensive histopathological, hematological, clinical chemistry, and genotoxicity assessments required for formal toxicological characterization.

The structural similarity of CBC to other well-characterized cannabinoids (THC, CBD) provides some basis for inference regarding expected toxicological endpoints. CBD, the most extensively studied non-psychoactive cannabinoid, has a favorable safety profile in humans at doses up to 1,500 mg/day, with hepatotoxicity (elevated transaminases) as the primary dose-limiting toxicity, particularly when co-administered with hepatotoxic drugs (e.g., valproate) (Kulpa et al. 2023; Taiwo et al. 2023). Whether CBC shares this hepatotoxic liability is unknown but plausible given the common CYP450-mediated hepatic metabolism. The reproductive and developmental toxicity of CBC has not been assessed, representing a critical gap for populations of childbearing potential.

Kulpa et al. (2023) provided the first dedicated subacute toxicological evaluation, administering four minor cannabinoids (including CBC) orally to rats for 14 days and assessing standard toxicological endpoints alongside pain measures (Kulpa et al. 2023). While not fully OECD-guideline-compliant, this study represents a meaningful advance beyond the incidental safety observations from pharmacology experiments. Additionally, Taiwo et al. (2023) investigated the reproductive toxicity of pre-puberty CBC exposure, finding that CBC modulated reproductive function via alteration of spermatogenesis, steroidogenesis, and eNOS pathway metabolites (Taiwo et al. 2023). These data partially address the reproductive toxicity knowledge gap and underscore the need for comprehensive developmental and reproductive toxicology (DART) studies prior to clinical advancement.

### Drug–drug interactions

The potential for pharmacokinetic drug–drug interactions (DDIs) mediated by CBC is a clinically relevant safety consideration. As discussed in Sect. "Metabolism", CBC inhibits multiple CYP450 isoforms (CYP3A4, CYP2C9, CYP2C19, CYP2D6) in vitro, and these enzymes collectively metabolize approximately 70–80% of clinically used drugs. The most significant interaction potential involves: CYP3A4 substrates (statins, immunosuppressants, protease inhibitors, certain chemotherapeutics), CYP2C9 substrates (warfarin, phenytoin, sulfonylureas), CYP2C19 substrates (proton pump inhibitors, clopidogrel, certain SSRIs), and CYP2D6 substrates (opioids, β-blockers, certain antidepressants) (Peters et al. 2022). However, as emphasized previously, the clinical relevance of these in vitro findings depends on the achievable plasma concentrations of CBC at therapeutic doses, which are expected to be substantially lower than the inhibitory concentrations reported in microsomal assays. Additionally, pharmacodynamic interactions, including additive sedation with CNS depressants, enhanced hypotension with antihypertensives, and potentiation of anticoagulant effects, represent theoretical concerns requiring clinical evaluation (Peters et al. 2022; Kulpa et al. 2023).

### Regulatory status

The regulatory classification of CBC varies significantly across jurisdictions and remains in flux as Cannabis legalization progresses. In the United States, CBC derived from hemp (*Cannabis sativa* containing less than 0.3% THC by dry weight) is arguably not a controlled substance under the 2018 Agriculture Improvement Act (Farm Bill), though the Food and Drug Administration (FDA) has not issued specific guidance on CBC. In the European Union, CBC is not explicitly listed in the EU Novel Food Catalogue, creating regulatory ambiguity for food and supplement applications. In jurisdictions with comprehensive medical Cannabis frameworks (Canada, Australia, Germany, Israel), CBC is generally accessible as a component of licensed Cannabis products but is not available as an approved pharmaceutical ingredient. The absence of a unified international regulatory framework for CBC complicates clinical development, cross-border trade, and product standardization. Regulatory harmonization, informed by comprehensive toxicological and clinical safety data, is essential for the advancement of CBC as a therapeutic agent.

## Clinical evidence and translational challenges

### Status of clinical trials

As of early 2026, no completed or ongoing clinical trials evaluating CBC as a single-agent therapeutic have been identified in the major clinical trial registries (ClinicalTrials.gov, EU Clinical Trials Register, WHO ICTRP). This complete absence of clinical data represents the most significant barrier to the therapeutic development of CBC and distinguishes it from other minor cannabinoids (CBG, CBN) for which early-phase clinical investigations have been initiated or completed. The sole published human pharmacokinetic data for CBC derive from the pilot study by (Peters et al. 2022), in which CBC was administered as a component of a multi-cannabinoid medical Cannabis product rather than as an isolated compound (Peters et al. 2022).

The absence of clinical trials reflects several interrelated barriers: (a) the historical overshadowing by THC and CBD research; (b) regulatory complexity surrounding Cannabis-derived products; (c) limited availability of pharmaceutical-grade, GMP-manufactured CBC for clinical use; (d) insufficient preclinical safety data to support Investigational New Drug (IND) applications; (e) intellectual property challenges, as CBC is a natural compound with limited patentability (though formulations and derivatives may be patentable); and (f) the difficulty of securing research funding for minor cannabinoid clinical trials in the current regulatory environment.

### Limitations in clinical translation

Beyond the absence of clinical trials, several pharmacological and practical challenges impede the clinical translation of CBC. The extremely low oral bioavailability necessitates either very high oral doses (with associated cost and compliance issues), alternative administration routes (inhalation, transdermal, sublingual), or advanced formulation strategies (nanotechnology, lipid-based delivery) to achieve therapeutically relevant plasma concentrations. The lack of validated biomarkers for CBC's pharmacodynamic effects complicates dose–response assessment in clinical settings. The potential for drug–drug interactions through CYP450 inhibition requires careful evaluation in patient populations likely to receive polypharmacy. The absence of standardized CBC products with defined purity, potency, and stability specifications limits the reproducibility and comparability of future clinical studies.

**Fig. 6:**
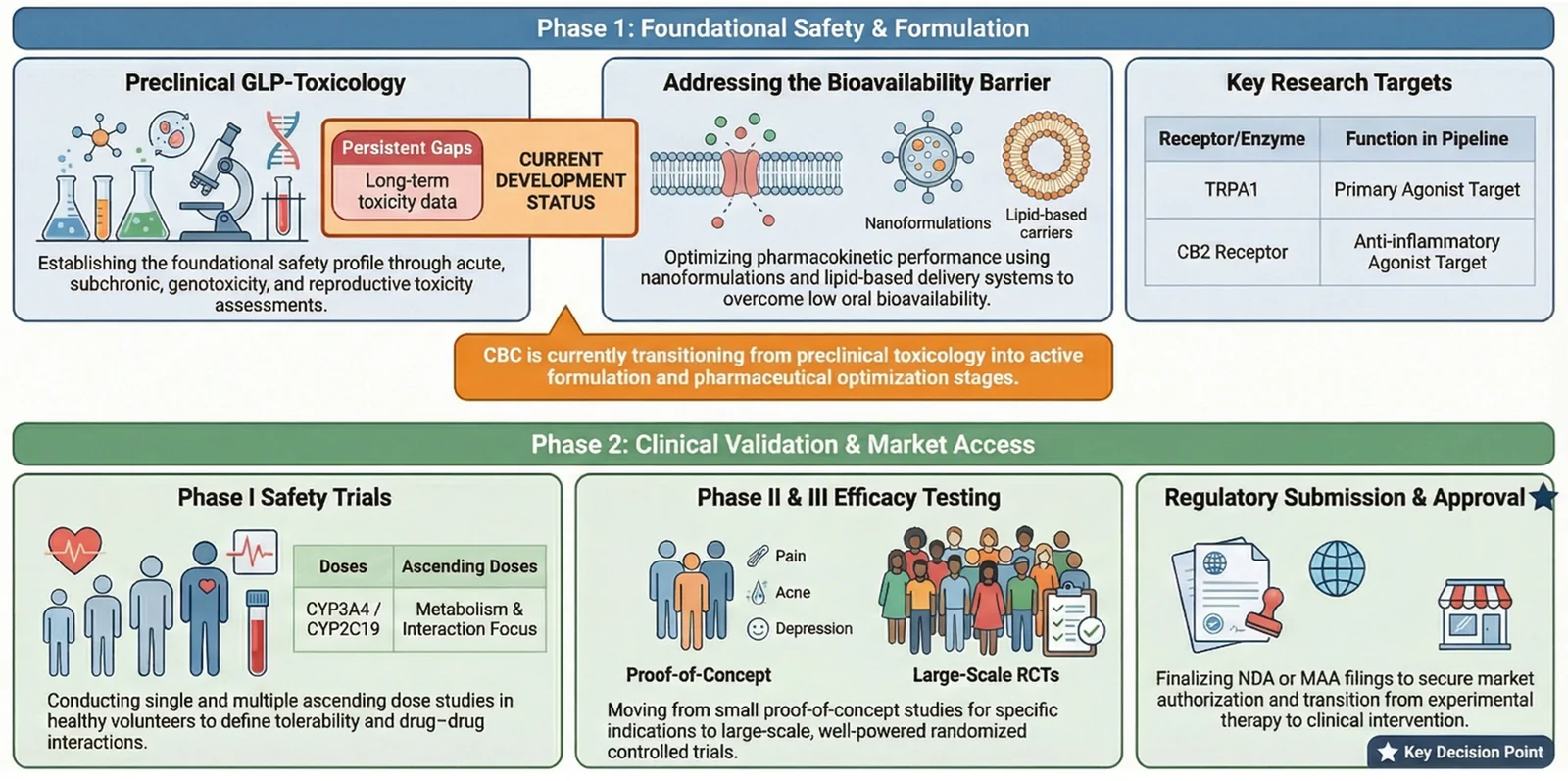
Proposed translational roadmap for CBC clinical development. CBC clinical development progresses through sequential stages, beginning with GLP toxicology (acute, subchronic, genotoxicity, reproductive toxicity), followed by formulation development (nanoformulations, enhanced bioavailability, stability testing). This is succeeded by Phase I trials (SAD/MAD, pharmacokinetics, drug–drug interaction studies), Phase IIa proof-of-concept studies for specific indications (pain, acne, depression), Phase IIb/III pivotal trials, and concludes with regulatory submission (NDA/MAA). Key decision points guide advancement, and gaps or unmet needs are identified at each stage

A structured clinical development roadmap for CBC should prioritize: (1) completion of GLP-compliant preclinical toxicology studies (acute, subchronic, chronic, genotoxicity, reproductive/developmental); (2) development of stable, well-characterized pharmaceutical formulations with enhanced bioavailability; (3) Phase I single ascending dose (SAD) and multiple ascending dose (MAD) studies in healthy volunteers to establish safety, tolerability, and pharmacokinetic profiles; (4) Phase Ib drug–drug interaction studies with common co-medications; (5) proof-of-concept Phase IIa studies in selected indications (e.g., inflammatory pain, acne vulgaris, or adjunctive antidepressant therapy) where the preclinical evidence is strongest; and (6) adequately powered Phase IIb/III randomized controlled trials with validated clinical endpoints (Fig. 6).

## Future perspectives

### Precision medicine and personalized cannabinoid therapy

The emergence of pharmacogenomics and precision medicine paradigms offers the prospect of individualized cannabinoid therapy, in which the selection and dosing of specific cannabinoids (including CBC) are tailored to the patient's genetic profile, disease phenotype, and co-medication regimen. Polymorphisms in CYP450 genes (CYP2C9, CYP2C19, CYP3A4) that influence cannabinoid metabolism, variations in TRP channel expression and function (TRPV1, TRPA1), and endocannabinoid system gene polymorphisms (CNR1, CNR2, FAAH, MGLL) may collectively determine individual responsiveness to CBC. The integration of pharmacogenomic testing with clinical CBC use could optimize therapeutic outcomes, minimize adverse effects, and identify patient subpopulations most likely to benefit from CBC-based interventions. This precision approach is particularly relevant for cancer therapy, where tumor-specific expression of TRP channels and cannabinoid receptors could guide the selection of CBC-containing regimens.

### Nanotechnology in CBC delivery

The application of nanotechnology to CBC delivery represents perhaps the most immediately impactful avenue for enhancing its therapeutic utility (Stasiłowicz-Krzemień et al. 2023). Beyond the formulation strategies discussed in Sect. "Stability and Formulation Considerations", emerging nanotechnology platforms including exosome-based delivery, metal–organic framework (MOF).

### Synthetic biology and sustainable production

The continued development of microbial and cell-free biosynthetic platforms for CBC production is essential for ensuring a sustainable, scalable, and regulatory-compliant supply chain (Luo et al. 2019; Qi et al. 2025; Schmidt et al. 2024; Kearsey et al. 2023; Love et al. 2024). Key priorities include: (a) protein engineering of CBCAS for enhanced catalytic efficiency, thermostability, and selectivity over competing synthases; (b) optimization of the entire biosynthetic pathway in yeast and bacterial hosts through dynamic metabolic regulation, compartmentalization strategies, and adaptive laboratory evolution; (c) development of plant-based production systems using transient or stable expression in tobacco (*Nicotiana benthamiana*) (Qian et al. 2019) or other non-Cannabis hosts; (d) exploration of cell-free enzymatic cascades that eliminate host toxicity and metabolic competition issues (Valliere et al. 2019); and (e) process engineering for scalable, GMP-compliant fermentation and purification.

### Multi-omics approaches

The integration of multi-omics technologies, genomics, transcriptomics, proteomics, metabolomics, and lipidomics, promises to deepen our understanding of CBC biosynthesis, mechanism of action, and therapeutic effects (Govindarajan et al. 2023; Lynch et al. 2016). Genome-wide association studies (GWAS) and quantitative trait locus (QTL) mapping in diverse Cannabis germplasm collections can identify novel genetic determinants of CBC accumulation beyond the known synthase genes (Govindarajan et al. 2023; Hazekamp et al. 2016; Lynch et al. 2016). Single-cell transcriptomics of glandular trichomes can resolve the cell-type-specific expression patterns of cannabinoid biosynthetic enzymes and regulatory factors. Untargeted metabolomics and lipidomics in CBC-treated cells and tissues can identify novel bioactive metabolites, endocannabinoid lipid species modulated by CBC, and downstream effectors of its pharmacological activity. Systems pharmacology approaches integrating multi-omics data with network-based computational models can generate mechanistic hypotheses for CBC's multi-target activity and guide rational combination therapy design (Sepulveda et al. 2024; Hwang et al. 2025).

### Regulatory harmonization

The fragmented and evolving regulatory landscape for CBC and other minor cannabinoids presents both challenges and opportunities. International regulatory harmonization is needed to (Calcaterra et al. 2023): (a) establish clear legal classification of CBC as a non-intoxicating, non-controlled substance distinct from THC; (b) develop pharmacopeial monographs and analytical specifications for CBC reference standards and finished products; (c) create streamlined regulatory pathways for CBC-based pharmaceutical products, nutraceuticals, and cosmeceuticals; (d) harmonize quality and safety standards for CBC-containing consumer products across major markets (North America, European Union, Asia–Pacific); and (e) facilitate the exchange of preclinical and clinical safety data across regulatory agencies to avoid unnecessary duplication of studies. The establishment of CBC-specific expert panels within existing regulatory bodies (FDA, EMA, TGA) and international organizations (WHO, ICH) would accelerate this process.

## Conclusions

Cannabichromene occupies a paradoxical position within the phytocannabinoid landscape: it is consistently among the most abundant cannabinoids in *Cannabis sativa*, ranking fourth when CBG is considered, was identified over half a century ago, possesses a unique and therapeutically relevant pharmacological profile, yet remains one of the most understudied members of its chemical class. This comprehensive review has consolidated the available evidence spanning biosynthesis, phytochemistry, pharmacokinetics, molecular pharmacology, preclinical efficacy, safety, and translational challenges, revealing both the substantial promise and the critical knowledge deficiencies that characterize the current state of CBC research. The biosynthetic origin of CBC through the CBCAS-catalyzed oxidative cyclization of CBGA is well-established, and advances in metabolic engineering and synthetic biology are beginning to provide sustainable production platforms independent of agricultural Cannabis cultivation. The chemical instability of CBC, particularly its photolability and low aqueous solubility, necessitates advanced formulation approaches that are technically feasible with current nanotechnology but require systematic optimization and validation for each intended route of administration.

The molecular pharmacology of CBC is distinguished by its potent multi-TRP channel agonism (TRPA1, TRPV1, TRPV3, TRPV4), minimal psychoactive potential, and indirect enhancement of endocannabinoid tone, a profile that is unique among phytocannabinoids and offers therapeutic mechanisms orthogonal to those of THC and CBD. The preclinical evidence base, while incomplete, supports multi-dimensional therapeutic potential across inflammation, pain, neurodegeneration, depression, cancer, skin disorders, and gastrointestinal conditions. The convergence of neuroprotective and neurogenic properties is particularly noteworthy and distinguishes CBC from most other cannabinoids and conventional pharmaceuticals.

However, the translation of this preclinical promise to clinical reality faces significant barriers. The complete absence of clinical trial data represents the most urgent deficiency, compounded by insufficient toxicological characterization, regulatory ambiguity, and pharmaceutical development challenges. The roadmap for clinical advancement of CBC must prioritize rigorous preclinical safety studies, development of bioavailability-enhanced formulations, and systematic Phase I–II clinical programs targeting indications where the mechanistic rationale and preclinical evidence are strongest.

In the broader context of phytocannabinoid pharmacology, CBC exemplifies the therapeutic opportunities that remain untapped within the minor cannabinoid repertoire. The reductionist focus on THC and CBD, while scientifically productive, has created a knowledge asymmetry that is no longer justified by the emerging pharmacological data. The next decade of cannabinoid research must embrace a more inclusive and systematic approach to the full cannabinoid spectrum, with CBC positioned as a priority candidate for accelerated investigation. The convergence of advanced analytical technologies, synthetic biology production platforms, nanotechnology delivery systems, multi-omics research tools, and evolving regulatory frameworks provides an unprecedented opportunity to translate the theoretical potential of CBC into validated therapeutic applications.

## Data Availability

No datasets were generated or analysed during the current study.

## Abbreviations

AEA: Anandamide (N-arachidonoylethanolamine)
ABTS: 2,2'-Azino-bis(3-ethylbenzothiazoline-6-sulfonic acid)
ASE: Accelerated solvent extraction
BBE: Berberine bridge enzyme
cAMP: Cyclic adenosine monophosphate
CB1R: Cannabinoid type 1 receptor
CB2R: Cannabinoid type 2 receptor
CBC: Cannabichromene
CBCA: Cannabichromenic acid
CBCAS: Cannabichromenic acid synthase
CBD: Cannabidiol
CBDA: Cannabidiolic acid
CBDAS: Cannabidiolic acid synthase
CBG: Cannabigerol
CBGA: Cannabigerolic acid
CBL: Cannabicyclol
CBN: Cannabinol
CFA: Complete Freund's adjuvant
COX-2: Cyclooxygenase-2
CPC: Centrifugal partition chromatography
CYP: Cytochrome P450
DDI: Drug-drug interaction
DMAPP: Dimethylallyl diphosphate
DPPH: 2,2-Diphenyl-1-picrylhydrazyl
DSS: Dextran sodium sulfate
ECS: Endocannabinoid system
EVOO: Extra virgin olive oil
FAAH: Fatty acid amide hydrolase
FAD: Flavin adenine dinucleotide
FST: Forced swim test
GC-MS: Gas chromatography-mass spectrometry
GLP: Good laboratory practice
GMP: Good manufacturing practice
GPP: Geranyl diphosphate
GPPS: Geranyl diphosphate synthase
GRAS: Generally recognized as safe
GWAS: Genome-wide association study
HO-1: Heme oxygenase-1
HPLC-DAD: High-performance liquid chromatography with diode array detection
HP-beta-CD: Hydroxypropyl-beta-cyclodextrin
IBD: Inflammatory bowel disease
IKK: IκB kinase
iNOS: Inducible nitric oxide synthase
IPP: Isopentenyl diphosphate
LC–MS/MS: Liquid chromatography-tandem mass spectrometry
LPS: Lipopolysaccharide
MAE: Microwave-assisted extraction
MAGL: Monoacylglycerol lipase
MEP: Methylerythritol phosphate
MIC: Minimum inhibitory concentration
MMP: Matrix metalloproteinase
MOF: Metal-organic framework
MRM: Multiple reaction monitoring
MRSA: Methicillin-resistant *Staphylococcus aureus*
NADES: Natural deep eutectic solvent
NF-κB: Nuclear factor kappa-light-chain-enhancer of activated B cells
NLC: Nanostructured lipid carrier
NMR: Nuclear magnetic resonance
NO: Nitric oxide
Nrf2: Nuclear factor erythroid 2-related factor 2
NSAID: Non-steroidal anti-inflammatory drug
NSPC: Neural stem/progenitor cell
OA: Olivetolic acid
OAC: Olivetolic acid cyclase
OLS: Olivetol synthase
PCL: Polycaprolactone
PGE2: Prostaglandin E2
PLE: Pressurized liquid extraction
PLGA: Poly(lactic-co-glycolic acid)
PPAR: Peroxisome proliferator-activated receptor
QTL: Quantitative trait locus
RNS: Reactive nitrogen species
ROS: Reactive oxygen species
SAR: Structure-activity relationship
SC-CO_2_: Supercritical carbon dioxide
SEDDS: Self-emulsifying drug delivery system
SLN: Solid lipid nanoparticle
SNEDDS: Self-nanoemulsifying drug delivery system
SOD: Superoxide dismutase
STAT3: Signal transducer and activator of transcription 3
THC: Delta-9-tetrahydrocannabinol
THCA: Tetrahydrocannabinolic acid
THCAS: Tetrahydrocannabinolic acid synthase
TKS: Tetraketide synthase
TRP: Transient receptor potential
TRPA1: Transient receptor potential ankyrin 1
TRPM8: Transient receptor potential melastatin 8
TRPV: Transient receptor potential vanilloid
TST: Tail suspension test
UAE: Ultrasound-assisted extraction
UGT: UDP-glucuronosyltransferase
